# Bortezomib‐Encapsulated Dual Responsive Copolymeric Nanoparticles for Gallbladder Cancer Targeted Therapy

**DOI:** 10.1002/advs.202103895

**Published:** 2022-01-23

**Authors:** Mingyu Chen, Sarun Juengpanich, Shijie Li, Win Topatana, Ziyi Lu, Qiang Zheng, Jiasheng Cao, Jiahao Hu, Esther Chan, Lidan Hou, Jiang Chen, Fang Chen, Yu Liu, Sukanda Jiansirisomboon, Zhen Gu, Suparat Tongpeng, Xiujun Cai

**Affiliations:** ^1^ Department of General Surgery Sir Run‐Run Shaw Hospital Zhejiang University Hangzhou 310016 China; ^2^ School of Medicine Zhejiang University Hangzhou 310058 China; ^3^ College of Pharmaceutical Sciences Zhejiang University Hangzhou 310058 China; ^4^ School of Physical and Mathematical Sciences Nanyang Technological University Singapore 637371 Singapore; ^5^ Department of Chemistry Zhejiang University Hangzhou 310027 China; ^6^ College of Life Sciences Zhejiang University Hangzhou 310058 China; ^7^ School of Ceramic Engineering Institute of Engineering Suranaree University of Technology Nakhon Ratchasima 30000 Thailand

**Keywords:** drug delivery, gallbladder cancer, nanomedicine, proteasome inhibitor, targeted therapy

## Abstract

Gallbladder cancer (GBC) is a rare but the most malignant type of biliary tract tumor. It is usually diagnosed at an advanced stage and conventional treatments are unsatisfactory. As a proteasome inhibitor, bortezomib (BTZ) exhibits excellent antitumor ability in GBC. However, the long‐term treatment efficacy is limited by its resistance, poor stability, and high toxicity. Herein, BTZ‐encapsulated pH‐responsive copolymeric nanoparticles with estrone (ES‐NP_(BTZ; Ce6)_) for GBC‐specific targeted therapy is reported. Due to the high estrogen receptor expression in GBC, ES‐NP_(BTZ; Ce6)_ can rapidly enter the cells and accumulate near the nucleus via ES‐mediated endocytosis. Under acidic tumor microenvironment (TME) and 808 nm laser irradiation, BTZ is released and ROS is generated by Ce6 to destroy the “bounce‐back” response pathway proteins, such as DDI2 and p97, which can effectively inhibit proteasomes and increase apoptosis. Compared to the traditional treatment using BTZ monotherapy, ES‐NP_(BTZ; Ce6)_ can significantly impede disease progression at lower BTZ concentrations and improve its resistance. Moreover, ES‐NP_(BTZ; Ce6)_ demonstrates similar antitumor abilities in patient‐derived xenograft animal models and five other types of solid tumor cells, revealing its potential as a broad‐spectrum antitumor formulation.

## Graphical abstract

Tumor‐pH‐sensitive self‐assembled polymeric nanoparticles with estrone tumor‐targeting ligand for gallbladder cancer‐targeted treatment (bortezomib‐encapsulated nanoparticles with estrone ES‐NP_(BTZ; Ce6)_) are developed. Bortezomib inhibits the proteasomes, whereas the generated ROS damages the “bounce‐back” response proteins to inhibit new proteasome production. ES‐NP_(BTZ; Ce6)_ can effectively treat gallbladder cancer via enhanced proteasome dysfunction at reduced bortezomib concentrations while mitigating systemic toxicity both in vitro and in vivo.

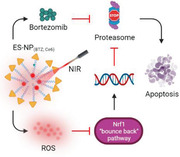

## 1 Introduction

Gallbladder cancer (GBC) is the most common biliary tract cancer with a poor prognosis.^[^ ^1^ ^]^ Due to the vague symptoms in the early stage, GBC is usually detected and diagnosed at an advanced stage whereby less than 30% of patients are eligible for curative surgical resection,^[^ ^2^ ^]^ resulting in a limited overall survival of 12–16 months. Even though surgical resection is one of the most effective treatments for GBC, more than 60% of patients suffer from postoperative recurrence within five years.^[^ ^3^ ^]^ Furthermore, conventional chemotherapy and radiotherapy are unable to significantly prolong overall survival in patients with advanced GBC. Recently, personalized targeted therapy that focuses on the ubiquitin‐proteasome system has emerged as a promising approach for advanced GBC treatment.^[^ ^4^ ^]^

The ubiquitin‐proteasome system is vital for cell survival since it eliminates defective proteins and prevents the accumulation of harmful proteins that can result in cell damage.^[^ ^5^ ^]^ Bortezomib (BTZ) is the first FDA‐approved proteasome inhibitor that exhibits significant antitumor activity in various tumors.^[^ ^6^ ^]^ However, BTZ usually leads to Nrf1 accumulation and migration to the cell nucleus through the “bounce‐back” response, mainly including retro‐translocation by ATPase p97 and DDI2 cleaving action to produce new proteasomes that trigger its resistance.^[^ ^7^ ^]^ In addition, the boric acid groups in BTZ can chelate with various reactive functional groups in the plasma protein and cause poor stability.^[^ ^8^ ^]^ Increasing BTZ concentrations can slightly improve the therapeutic effect, but with high toxicity. The long‐term treatment efficacy of BTZ is limited by its resistance, poor stability, and high toxicity.^[^ ^9^ ^]^ Therefore, a new approach with decreased toxicity and increased systemic stability for BTZ to solve its resistance is desired.

Herein, we have developed BTZ‐encapsulated, pH‐responsive, and NIR‐activated copolymeric nanoparticles with estrone (ES), ES‐PEG_2K_‐P(Asp‐DA)‐Ce6‐BTZ (ES‐NP_(BTZ; Ce6)_), for GBC‐targeted therapy. ES‐NP_(BTZ; Ce6)_ can rapidly enter GBC cells and aggregate near the nucleus via estrogen receptor (ER) and partially release BTZ and Ce6 under acidic tumor microenvironment (TME). BTZ inhibits the proteasomes, whereas the ROS generated by Ce6 after 808 nm laser irradiation damages a series of the “bounce‐back” response proteins such as DDI2 and p97, thereby inhibiting the production of new proteasomes that eventually lead to cell death. ES‐NP_(BTZ; Ce6)_ has various advantages in cancer therapy. For instance, ES acted as a tumor‐targeting ligand in GBC with relatively high ER expression,^[^ ^10^, ^11^ ^]^ to help nanoparticles rapidly enter the cells and accumulate near the nucleus via ES‐mediated endocytosis or tissue‐specific interactions.^[^ ^12^ ^]^ The acidic TME can also be exploited by tumor‐pH‐sensitive nanoparticles for controlled drug release.^[^ ^13^ ^]^ Moreover, this formulation, which consists of Chlorin e6 (Ce6) for photodynamic therapy (PDT), can react with molecular oxygen to eliminate tumor cells via oxidative stress,^[^ ^14^ ^]^ thus alleviating drug resistance by targeting the unique biochemical alterations or ROS‐mediated protein damage in the “bounce‐back” response.^[^ ^15^ ^]^

## 2 Results and Discussion

### 2.1 Synthesis and Characterization of ES‐NP_(BTZ; Ce6)_

The unique self‐assembly properties of block copolymer nanoparticles have the potential for drug delivery application developments. ES‐NP_(BTZ; Ce6)_ nanoparticle is a block copolymer that consists of hydrophilic and hydrophobic chains (Figure S1, Supporting Information). To synthesize the hydrophilic chain‐growth polymer (ES‐PEG_2K_‐COOH), ES is PEGylated with polyethylene glycol (PEG) and coupled with the carboxyl group from succinic acid as the cross‐linking moiety (Figure S1c, Supporting Information). PEG is generally fabricated with other polymers to form block PEG‐based copolymers, which have strong amphiphilicity and are expected to self‐assemble into nanoparticle structure in an aqueous solution. For hydrophobic chain (n‐butylamine‐P(Asp‐DA)) synthesis, polyaspartic acid was functionalized by ammonia reaction, followed by crosslinking the hydrophobic core with dopamine (DA) groups to increase its stability. Ammonia solution is generally used to modify the poly(*β*‐benzyl‐_L_‐aspartate) (PBLA) of the main‐chain polymer, which was converted to the amino group for the preparation of drug‐containing conjugates (Figure S1d, Supporting Information). If the carrier has an amino group as a preferred modifier, it can provide an active site for subsequent polymer–drug encapsulation reactions. To enhance the pH sensitivity of the polymer, the catechol moiety was introduced into the hydrophobic chain. The boronic acid group in the BTZ structure is mainly responsible for the reaction with dopamine (DA) by establishing a boronate ester bond between BTZ and catechol, and Ce6 was loaded into the main‐chain polymer via the amino group of the hydrophobic chain and the carboxyl group of Ce6 (Figure S1e,f, Supporting Information). This interaction also has pH‐dependent properties to cleavage in acidic conditions, ensuring the release of BTZ in acidic tumor tissue.

**Figure 1:**
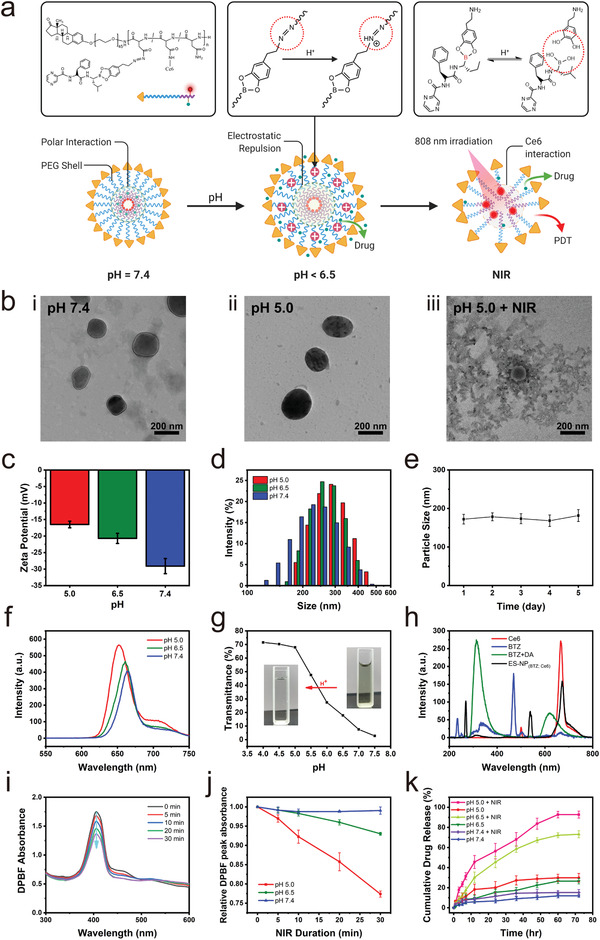
Characterization of ES‐NP_(BTZ; Ce6)_. a) Schematic illustration of tumor pH activation and NIR irradiation of ES‐NP_(BTZ; Ce6)_. b) TEM image of ES‐NP_(BTZ; Ce6)_ at pH 7.4 (i), pH 5.0 (ii), and pH 5.0 after 808 nm laser irradiation (iii). c) Zeta potential changes of ES‐NP_(BTZ; Ce6)_ at different pH. d) ES‐NP_(BTZ; Ce6)_ particle size distribution at different pH. e) Stability test of ES‐NP_(BTZ; Ce6)_ in PBS via DLS measurements. f) Ce6 fluorescence intensity from ES‐NP_(BTZ; Ce6)_ at different pH (Excitation = 400 nm, Emission = 660 nm). g) pH‐dependent transmittance changes of ES‐NP_(BTZ; Ce6)_. h) Fluorescence intensity of Ce6, BTZ, BTZ+DA, and ES‐NP_(BTZ; Ce6)_. i) ES‐NP_(BTZ; Ce6)_ DPBF absorbance spectrum changes at pH 5.0 under 808 nm laser irradiation over multiple time intervals. j) ES‐NP_(BTZ; Ce6)_ pH‐dependent DPBF absorbance under 808 nm laser irradiation at different pH. k) In vitro drug release profile of ES‐NP_(BTZ; Ce6)_. The data are represented as mean ± SD (*n* = 3).

As shown in **Figure** **1**a, the hydrophobic chain is pH‐responsive, in which the change in pH value will result in the exchange of protons in the aqueous solution, thereby altering the hydrophilicity of the related chain. To formulate ES‐NP_(BTZ; Ce6)_ and encapsulate drugs, BTZ and Ce6 were loaded into the hydrophobic core and self‐assembled with the hydrophilic shell. Due to the high ER expression in GBC (Figure S2, Supporting Information), the surface‐exposed estrone in the hydrophilic chain act as a tumor‐targeting ligand,^[^ ^10^, ^11^, ^16^ ^]^ whereas the internally packed DA in the hydrophobic chain serves as an ionizable moiety for pH sensitivity.^[^ ^17^ ^]^ The use of hydroxyl group as a pH‐sensitive ionizable link between the encapsulated drugs ensures targeted drug delivery at acidic TME, thus lowering drug concentration and avoiding unwanted adverse effects. Moreover, NIR‐activation also serves as a second layer of safeguard for precise cancer treatment.

The successful conjugation of ES‐NP_(BTZ; Ce6)_ was confirmed via ^1^H NMR in Figures S3–S6 (Supporting Information). The peaks at *δ* = 2.7, 2.8, 3.3, 4.3, 5.3, 5.5, 6.6, 7.2, and 7.9 ppm were attributed to the protons of methylene groups in the PEG block and aromatic of ES (Figure S4, Supporting Information). Additionally, signals at *δ* = 0.8, 1.0–1.3, 2.0, 2.7, 3.2, 4.4, 5.2, 7.3, and 7.9 ppm were attributed to hydrophobic chain protons and phenyl protons of DA blocks (Figure S5, Supporting Information). As depicted in Figure S6 (Supporting Information), all hydrophilic and hydrophobic chains peaks were detected in the ^1^H NMR spectra, demonstrating that the carrier was successfully fabricated. Finally, the new peak at 9–10 ppm was attributed to the protons of BTZ and Ce6, indicating that BTZ and Ce6 were successfully conjugated to the main polymer. Furthermore, the hydrophobic chains were further confirmed by FTIR and Raman spectrometry (Figure S7, Supporting Information). The aromatic C−O stretching, C−C stretching vibrations, and C−H stretching bands at 1213, 1411, and 2955 cm^–1^, respectively, confirmed the success of DA reactions and the formation of P(Asp‐DA) intermediates. The Raman shift peak at 1608 cm^–1^ corresponded to the stretching vibration absorbance of N═N of the amide group. The spectra revealed a signal band at approximately 3200–3500 cm^–1^, which corresponded to the stretching vibration absorbance of the hydrophobic chain O−H and N−H bonds. In addition, Figure S8 (Supporting Information) depicts the UV–visible spectra of Ce6, BTZ, BTZ‐DA, and ES‐NP_(BTZ; Ce6)_. The peak near 280 nm is BTZ, while the peaks near 410 and 660 nm are Ce6 absorption peaks. The characteristic peaks of BTZ and Ce6 were detected simultaneously in the UV spectra of ES‐NP_(BTZ; Ce6)_, indicating successful drug encapsulation. All of these results demonstrated that the polymer conjugation and synthesis of ES‐NP_(BTZ; Ce6)_ nanoparticles were successful.

To verify ES‐NP_(BTZ; Ce6)_ response to pH and NIR‐activation, transmission electron microscopy (TEM) (Figure 1b) has been employed to characterize the microstructures of the polymeric nanoparticles. ES‐NP_(BTZ; Ce6)_ is negatively charged (‐29.1 mV) and has an average hydrodynamic diameter of ≈180 nm at pH 7.4 (Figure 1c,d), which remained stable for at least 5 d (Figure 1e), indicating the high stability of the nanoparticles. However, pH changes lead to the formation of spherical morphology due to the osmotic swelling property of the polymeric nanoparticles, resulting in a slightly less negative surface charge (‐20.7 mV) and an increased average hydrodynamic diameter of ≈230 nm at acidic pH 6.5, with similar trends observed at pH 5.0 (‐16.5 mV; ≈260 nm). The practical applications of ES‐NP_(BTZ; Ce6)_ are unlimited owing to their structural stability, which can endure dilution or external condition changes such as changes in pH, solvent type, and ionic strength. Upon NIR irradiation, the TEM image revealed that ES‐NP_(BTZ; Ce6)_ disintegrated and became shapeless due to the unstable structure at acid pH.

At neutral pH 7.4, the photoactivity and fluorescence intensity of Ce6 in ES‐NP_(BTZ; Ce6)_ is substantially quenched owing to its proximity between one another. However, as the pH decreases from 7.4 to 5.0, ES‐NP_(BTZ; Ce6)_ starts to expand and the distance between Ce6 widens, leading to the recovery of both Ce6 photoactivity and fluorescence activity in ES‐NP_(BTZ; Ce6)_, thereby increasing ES‐NP_(BTZ; Ce6)_ fluorescence intensity (Figure 1f). The increase in ES‐NP_(BTZ; Ce6)_ transmittance at acidic pH results from the hydrogen ions reacting with the N═N bonds on the cross‐linked hydrophobic chains by accepting protons, which results in its expansion and the slight shift in surface charge (Figure 1g). In addition, boric acid can crosslink dopamine‐modified P(Asp), which yields the hydrophobic chain due to the boron–catechol coordination. The boronic acid interaction between BTZ and DA can be oxidized and hydrolyzed upon H_2_O_2_ exposure in the acidic TME,^[^ ^18^ ^]^ leading to the dissociation of ES‐NP_(BTZ; Ce6)_. ES‐NP_(BTZ; Ce6)_ typically exhibits an identical response to pH and ionic strength, thus a higher swelling ratio can be achieved by increasing ionic strength or decreasing pH, resulting in the cationic repulsion of ES‐NP_(BTZ; Ce6)_. This demonstrates the excellent ability of ES‐NP_(BTZ; Ce6)_ to undergo a reversible transition from clear to a turbid solution in response to pH change.

Fluorescence spectroscopy was used to verify the interaction between polymer, BTZ, and Ce6, and to estimate the encapsulation efficiency. According to the results, the peaks of ES‐NP_(BTZ; Ce6)_ at approximately 420 and 670 nm correspond to BTZ and Ce6, respectively (Figure 1h). When dissolved in PBS, the BTZ peak presents a strong fluorescence emission at *λ*_em_ = 420 nm after excitation at a wavelength *λ*_ex_ = 265 nm. Both BTZ and Ce6 peaks were detected simultaneously from ES‐NP_(BTZ; Ce6)_ in UV–visible (Figure S8, Supporting Information) and fluorescence spectrum (Figure S9, Supporting Information), indicating successful modification. Based on the HPLC results, ES‐NP_(BTZ; Ce6)_ has BTZ and Ce6 loading efficiency of 85% and 94%, respectively, and a drug‐to‐carrier ratio of 4 wt% (Figure S10, Supporting Information). Moreover, the pH‐dependent production of ROS induced by Ce6 upon near‐infrared (NIR) irradiation was investigated using a singlet oxygen (^1^O_2_) indicator, 1,3‐diphenylisobenzofuran (DPBF), in which ^1^O_2_ irreversibly quenches its absorption.^[^ ^19^ ^]^ As depicted in Figure 1h, the emission peaks of Ce6 molecules match with the peaks of ES‐NP_(BTZ; Ce6)_, demonstrating the usage of ES‐NP_(BTZ; Ce6)_ as a transducer for the activation of Ce6. Under acidic pH 6.5 and 5.0, the DPBF absorption intensity decreased with respect to NIR irradiation duration (Figure 1i,j), suggesting that ^1^O_2_ was efficiently generated by Ce6. In contrast, the DPBF absorption intensity in physiological pH 7.4 exhibited a minimal decrease under NIR irradiation (Figure S11, Supporting Information).

ES‐NP_(BTZ; Ce6)_ was further examined via in vitro drug release using the reverse dialysis method. ES‐NP_(BTZ; Ce6)_ is a dual responsive copolymeric nanoparticle that is responsive to acidic conditions to partially release the encapsulated drugs and has to be activated with NIR irradiation to release the remaining drugs. The drugs are released from the polymeric nanoparticles through the degradation or expansion of the structure as a result of pH changes. BTZ was not released under NIR irradiation without acidic conditions and is partially released at acidic pH. As shown in Figure 1k, BTZ were sparsely released (11.8%) at neutral pH 7.4 and partially released (pH 6.5, 26.5%; pH 5.0, 29.8%) at acidic pH after 72 h. This is due to ES‐NP_(BTZ; Ce6)_ response to acidic pH, in which hydrophobic and ionic interactions between BTZ and the copolymer system loosened ES‐NP_(BTZ; Ce6)_ structure, thereby partially releasing BTZ. Meanwhile, the intact structure of ES‐NP_(BTZ; Ce6)_ before NIR irradiation keeps BTZ from being completely released. Furthermore, NIR irradiation of ES‐NP_(BTZ; Ce6)_ at pH 7.4 did not result in particle disintegration, with a slight release of BTZ (15.1%), suggesting the excellent stability of ES‐NP_(BTZ; Ce6)_ structure at neutral pH that prevents BTZ from being released under NIR irradiation. Subsequently, NIR irradiation with 808 nm laser under acidic conditions to activate the Ce6 molecules will lead to the complete disintegration of ES‐NP_(BTZ; Ce6)_ to release all residual drugs (pH 6.5, 73.2%; pH 5.0, 92.7%). The dual responsive nature of ES‐NP_(BTZ; Ce6)_ was designed to ensure a safe and precise treatment, in which NIR‐activation serves as a second layer of safeguard for targeted cancer therapy.

### 2.2 In Vitro GBC Cellular Uptake and Cytotoxicity of ES‐NP_(BTZ; Ce6)_

**Figure 2:**
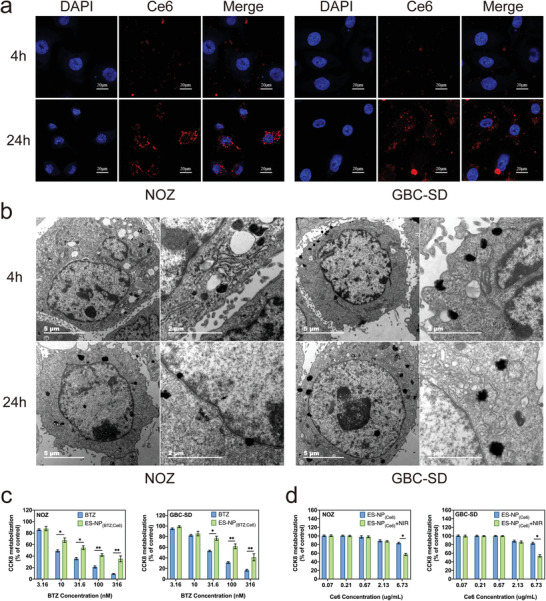
ES‐NP_(BTZ; Ce6)_ interactions with GBC cells. a) CLSM images to demonstrate ES‐NP_(BTZ; Ce6)_ cellular uptake by NOZ or GBC‐SD cells at different time points under acidic TME (pH 6.5). DAPI‐stained cells are shown in blue; ES‐NP_(BTZ; Ce6)_ fluorescence signals are shown in red. b) Cyro‐TEM images of ES‐NP_(BTZ; Ce6)_ cellular uptake by NOZ or GBC‐SD cells at different time points under acidic TME (pH 6.5). c) 48 h CCK‐8 assays of NOZ or GBC‐SD cells treated with different BTZ (equivalent to the dose loaded in ES‐NP_(BTZ; Ce6)_) or ES‐NP_(BTZ; Ce6)_ concentrations. d) 48 h CCK‐8 assays of NOZ or GBC‐SD cells exposed to different concentrations of ES‐NP_(Ce6)_ or ES‐NP_(Ce6)_ with 808 nm laser irradiation (2 W cm^–2^, 5 min with every min interval). The data are represented as mean ± SD (*n* = 3). *P < 0.05, **P < 0.01.

As depicted in confocal laser scanning microscopy (CLSM) imaging, ES‐NP_(BTZ; Ce6)_ accumulated around the nucleus of both GBC cell lines (NOZ and GBC‐SD) (**Figure** **2**a; Figure S12, Supporting Information). Due to the increased level of Ce6 release resulting from the expansion of ES‐NP_(BTZ; Ce6)_ under acidic pH that leads to the recovery of both Ce6 photoactivity and fluorescence activity, there is a higher fluorescence intensity in acidic pH 6.5 than neutral pH 7.4. Therefore, the existence of Ce6 signals from ES‐NP_(BTZ; Ce6)_ around DAPI‐stained cells in CLSM demonstrated higher cellular uptake at pH 6.5. By following ES‐NP_(BTZ; Ce6)_ with TEM, we found that ES‐NP_(BTZ; Ce6)_ could be quickly endocytosed into the cell. Once ES‐NP_(BTZ; Ce6)_ interacts with the acidic TME, it can gradually enlarge and accumulate near the cell nucleus membrane, which was consistent with CLSM imaging results (Figure 2b). This suggests that the estrone on ES‐NP_(BTZ; Ce6)_ can specifically bind with the ER on the cell and nucleus membrane of NOZ and GBC‐SD cells, which effectively drives the endocytosis and nucleus accumulation of ES‐NP_(BTZ; Ce6)_.

Furthermore, the cytotoxicity of ES‐NP_(BTZ; Ce6)_ and the encapsulated Ce6 under NIR irradiation have been investigated. No additional cytotoxicity was observed in ES‐NP_(BTZ; Ce6)_ compared with BTZ monotherapy at an equal concentration in both GBC cell lines and human intrahepatic biliary epithelial cell (HIBEC) line (Figure 2c; Figure S13, Supporting Information). The antiproliferative effects of ES‐NP_(BTZ; Ce6)_ were slightly lower than BTZ, resulting from the slow and incomplete drug release rate without laser irradiation. In addition, the concentration of Ce6 has been meticulously chosen to not be harmful to the cells. At equal Ce6 dose in ES‐NP_(BTZ; Ce6)_, no cell‐killing effects in the absence of BTZ have been observed, even when combined with NIR irradiation (Figure 2d).

### 2.3 Inhibition of New Proteasome Production by Breaking the “Bounce‐Back” Response

**Figure 3:**
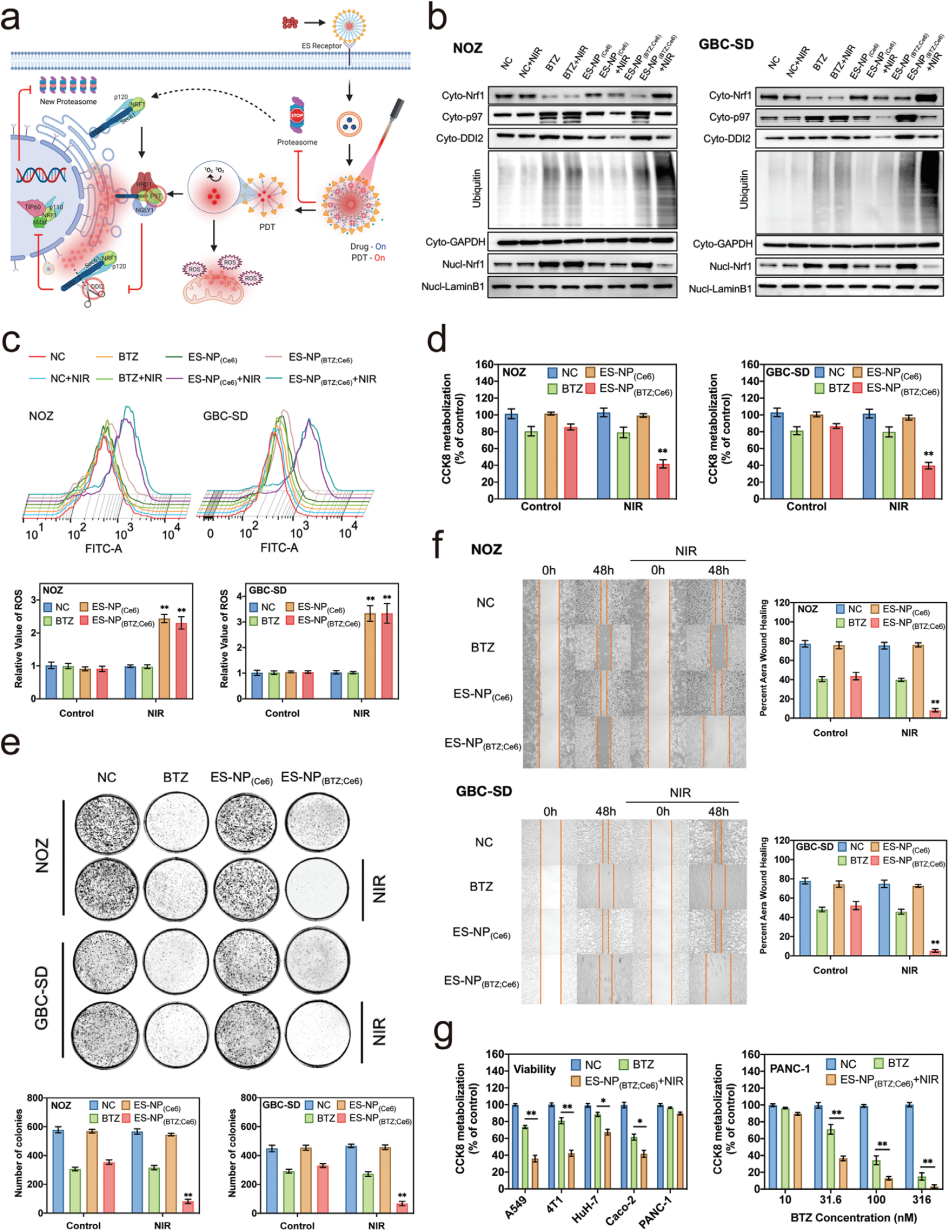
In vitro antitumor activity of ES‐NP_(BTZ; Ce6)_. a) Schematic illustration of the molecular mechanism underlying effects of ES‐NP_(BTZ; Ce6)_. b) Western blot for Nrf1, p97, DDI2, and ubiquitin in the cytoplasm or/and nucleus of NOZ or GBC‐SD cells exposed to normal media, BTZ, ES‐NP_(Ce6)_, or ES‐NP_(BTZ; Ce6)_ with/without 808 nm laser irradiation (2 W cm^–2^, 5 min with every min interval). c) ROS assays of NOZ or GBC‐SD cells treated as indicated by flow cytometry. d) 48 h CCK‐8 assays of NOZ or GBC‐SD cells exposed to normal media, BTZ, ES‐NP_(Ce6)_, or ES‐NP_(BTZ; Ce6)_ with/without 808 nm laser irradiation (2 W cm^–2^, 5 min with every min interval). e) Representative images of colonies formed by NOZ or GBC‐SD cells treated as specified and measurement of colony numbers in treatment groups. f) Representative images of cell mobility in NOZ or GBC‐SD cells treated as specified and measurement of wound‐healing assays. g) Left: 48 h CCK‐8 assays of various cancer cells exposed to the same concentration of BTZ (10 × 10^‐9^ m, equivalent to the dose loaded in ES‐NP_(BTZ; Ce6)_) or ES‐NP_(BTZ; Ce6)_ with 808 nm laser irradiation (2 W cm^–2^, 5 min with every min interval). Right: 48 h CCK‐8 assays of more concentrations 31.6 × 10^‐9^, 100 × 10^‐9^, 316 × 10^‐9^ m (in terms of BTZ dose) for PANC‐1. The data are represented as mean ± SD (*n* = 3). *P < 0.05, **P < 0.01.

Based on the aforementioned studies, our ES‐NP_(BTZ; Ce6)_ has a strong tumor‐killing ability theoretically through the synergistic effect, as it could release BTZ to destroy proteasomes and simultaneously prevent the tumor cells from compensating proteasomes to avoid drug resistance (**Figure** **3**a). The specific treatment mechanism of ES‐NP_(BTZ; Ce6)_ was further explored and validated in GBC cell lines (NOZ and GBC‐SD). As shown in Figure 3b, ubiquitin expression increased after BTZ administration, leading to decreased cytoplasm‐Nrf1 and increased nucleus‐Nrf1 to resupply proteasomes, which primarily originated from Nrf1 accumulation and migration to the cell nucleus through an array of biochemical reactions, including retro‐translocation by ATPase p97 and DDI2 cleaving action (Figure S14, Supporting Information). Our ES‐NP_(BTZ; Ce6)_ could release BTZ for targeted inhibition of proteasomes, but also generate ROS for protein and oxidative stress damage. In brief, the tumor‐killing effect of our nanoparticles is mostly due to BTZ's ability to inhibit proteasomes, while ROS serves as a supporting role. For example, under 808 nm NIR laser irradiation, ROS generated by ES‐NP_(BTZ; Ce6)_ could damage the DDI2 and p97 (also presented in ES‐NP_(Ce6)_+NIR), resulting in the inhibition of the proteasomes “bounce back” with a higher expression of ubiquitin in the cells than BTZ monotherapy, which revealed that its drug resistance was reversed, thus significantly increasing tumor cells apoptosis.

Of note, ROS generated by ES‐NP_(BTZ; Ce6)_ had minimal influence on ubiquitin. Ubiquitin is a heat‐stable polypeptide that could resist photodynamic or ROS‐medicated protein damage.^[^ ^20^ ^]^ Most importantly, the concentrations of Ce6 in ES‐NP_(Ce6)_ or ES‐NP_(BTZ; Ce6)_ used in the GBC cell lines (about 0.1 µg mL^‐1^) generated relatively low ROS levels at around two to fourfold, which is significantly lower than that of dependent ROS generated by Ce6 for direct tumor treatment via oxidative stress (Figure 3c; Figure S15, Supporting Information). To achieve tumor‐pH‐sensitive photodynamic nanoagents (PPNs) significant tumor‐killing effects after NIR irradiation, Ce6 concentration in the PPNs is around 3–6 µg mL^‐1^,^[^ ^21^ ^]^ which is substantially higher than our ES‐NP_(BTZ; Ce6)_. Additionally, proteasomes are widely distributed throughout the cell and our ES‐NP_(BTZ; Ce6)_ mainly accumulates around the nucleus, only a small amount of the proteasomes around the nucleus are destroyed after 808 nm laser irradiation, thus having little influence on the expression of total ubiquitin in the cell.

### 2.4 In Vitro Antitumor Activity of ES‐NP_(BTZ; Ce6)_

Proliferation assessments in GBC cell lines (NOZ and GBC‐SD) were performed to evaluate the synergetic effects of our ES‐NP_(BTZ; Ce6)_+NIR, using a Cell Counting Kit‐8 (CCK‐8) assay. The half‐maximal inhibitory concentration (IC50) of ES‐NP_(BTZ; Ce6)_+NIR against NOZ cells is 4.8 × 10^‐9^ m (in terms of BTZ dose), a 2.4‐fold decrease, compared with 11.4 × 10^‐9^ m for BTZ alone (Figure S16, Supporting Information). A similar result was observed in GBC‐SD. Next, the BTZ concentrations below IC50 (5 × 10^‐9^ m for NOZ; 10 × 10^‐9^ m for GBC‐SD) were selected to further validate the synergetic effects and antitumor activity of ES‐NP_(BTZ; Ce6)_ (Figure S17, Supporting Information). Equal concentration of ES‐NP_(BTZ; Ce6)_ exhibited relatively lower cytotoxicity than free BTZ, demonstrating the excellent biocompatibility and the controlled release property of the carriers. Notably, significant antiproliferative effects were observed in the ES‐NP_(BTZ; Ce6)_ group upon NIR irradiation, and the cells treated with drug‐free ES‐NP_(Ce6)_ did not show significant inhibition of proliferation, indicating the effectiveness of BTZ‐PDT (Figure 3d). The 72 h growth curve of NOZ or GBC‐SD also presented a similar result in ES‐NP_(BTZ; Ce6)_+NIR with the most significant antiproliferative effects (Figure S18, Supporting Information). Likewise, apoptosis conditions measured by flow cytometry were identical to that of antiproliferation assessments (Figure S19, Supporting Information). Hence, the synergetic effects between BTZ and NIR were only observed in ES‐NP_(BTZ; Ce6)_. Additionally, both cell colony‐forming and migration abilities of the tumors were significantly suppressed in cells treated by ES‐NP_(BTZ; Ce6)_+NIR, which further validated the antiproliferative results (Figure 3e,f).

Furthermore, the role of ES as an active targeting ligand in ES‐NP_(BTZ; Ce6)_ was evaluated in vitro. As shown in Figure S20 (Supporting Information), ES‐NP_(BTZ; Ce6)_+NIR inhibited cell proliferation in both NOZ and GBC‐SD cells with IC50 values of approximately 4.1× 10^‐9^ and 9.2 × 10^‐9^ m (in terms of BTZ dose), respectively. The IC50 was lower than that of NP_(BTZ; Ce6)_+NIR in NOZ (6.9 × 10^‐9^ m) and GBC‐SD (15.3 × 10^‐9^ m), which revealed that our nanoparticles with ES could rapidly target proteasome inhibition for enhanced therapeutic efficiency. Due to the promising antitumor activity of ES‐NP_(BTZ; Ce6)_+NIR in vitro, we further validated it on other solid tumors, including breast cancer (4T1), colon cancer (Caco‐2), liver cancer (HuH‐7), lung cancer (A549), and pancreatic cancer (PANC‐1) cells. Although the therapeutic efficiency of BTZ (10 × 10^‐9^ m) varied between each cancer cell, ES‐NP_(BTZ; Ce6)_+NIR (at the same dose of BTZ) always showed a stronger inhibition of cell proliferation than that of BTZ monotherapy across varied cell lines (Figure 3g). The therapeutic efficiency of BTZ depended on the sensitivity of the ubiquitin‐proteasome system. For example, PTEN‐intact cancer cell lines are less susceptible to proteasome inhibitors than PTEN‐mutant cancers, thus requiring a higher dose of BTZ.^[^ ^22^ ^]^ However, ES‐NP_(BTZ; Ce6)_ could partly overcome its resistance by incorporating PDT to achieve the synergistic treatment effect in various cancers under a lower dose of BTZ.

### 2.5 In Vivo Antitumor Activity of ES‐NP_(BTZ; Ce6)_

**Figure 4:**
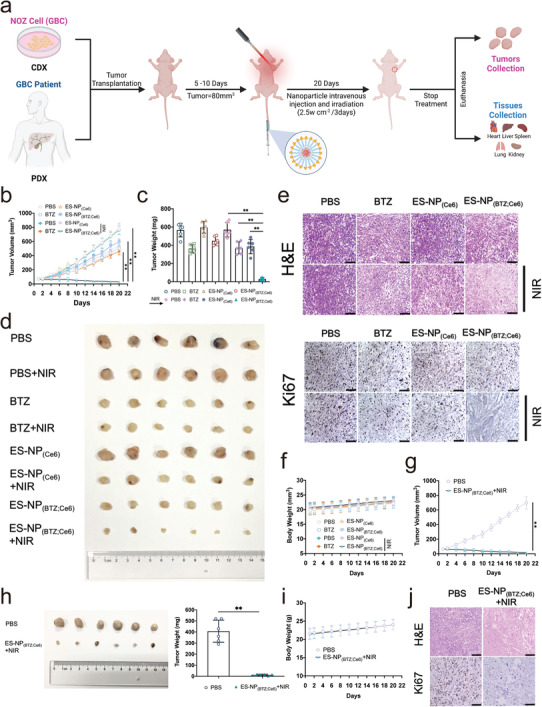
In vivo antitumor therapeutic effect of ES‐NP_(BTZ; Ce6)_. a) Schematic illustration of CDX or PDX animal model establishment and treatment. b) Tumor volume changes in the specified groups during treatment of CDX model. c) Statistical plot of the average tumor weights of the CDX model. d) Tumor images after 20 d of treatment in different groups. e) Representative images of H&E and Ki67 staining of collected tumor sections from different CDX model groups at 20 d posttreatment. f) Mice body weight changes in different groups of CDX model. g) Tumor volume changes in the specified groups during PDX model treatment. h) Tumor images and tumor weights in the specified groups of PDX model after 20 d posttreatment. i) Mice body weight changes in different groups of PDX model. j) Representative images of H&E and Ki67 staining of collected tumor sections from different PDX model groups at 20 d posttreatment. The data are represented as mean ± SD (*n* = 6 mice per group). **P < 0.01; scale bar = 100 µm.

We further evaluated the synergetic antitumor efficacy of ES‐NP_(BTZ; Ce6)_ on GBC cell‐derived xenograft (CDX) and patient‐derived xenograft (PDX) animal models (**Figure** **4**a; Figures S21 and S22, Supporting Information). Different formulations, including PBS, BTZ, ES‐NP_(Ce6)_, and ES‐NP_(BTZ; Ce6)_, were intravenously injected every 3 days for a total of 6 times at the same dose of BTZ (0.25 mg kg^–1^) or Ce6 (5.1 mg kg^–1^) and combined with or without NIR at a power density of 2.5 W cm^–2^ for 10 min, with 1 min interval after each minute of irradiation. As shown in Figure 4b, compared with PBS, every group (BTZ, BTZ +NIR, ES‐NP_(Ce6)_+NIR, ES‐NP_(BTZ; Ce6)_, and ES‐NP_(BTZ; Ce6)_+NIR), except PBS+NIR and ES‐NP_(Ce6)_, led to significant tumor volume growth inhibition. Notably, ES‐NP_(BTZ; Ce6)_+NIR exhibited superior antitumor efficacy throughout the experimental period, leading to a reduction of 96.8 ± 1.5% in tumor volume after treatment, which was substantially superior to that of BTZ (39.4 ± 5.9%), ES‐NP_(Ce6)_+NIR (29.6 ± 6.7%), and ES‐NP_(BTZ; Ce6)_ (27.4 ± 4.2%). The mice were euthanized after 20 d of treatment, and the excised tumors were photographed and weighed, with similar findings as tumor volume (Figure 4c,d). Subsequently, histopathological analysis using hematoxylin and eosin (H&E) staining of the tumors tissue sections revealed numerous apoptotic cells, decreased cellularity, and damaged cancer cells in the ES‐NP_(BTZ; Ce6)_+NIR group. Likewise, Ki‐67 immunolabeling revealed obvious inhibition of cancer cells proliferation in the same group, which further confirmed the excellent antitumor capability of ES‐NP_(BTZ; Ce6)_+NIR. Additionally, weight loss was not detected in mice injected with ES‐NP_(BTZ; Ce6)_ followed by irradiation, demonstrating that ES‐NP_(BTZ; Ce6)_ has minimal systemic toxicity (Figure 4f). Furthermore, a similar therapeutic efficacy of ES‐NP_(BTZ; Ce6)_+NIR was observed and verified on the PDX animal model that originated from an advanced GBC patient (Figure 4g–j). For deep‐located GBC, our nanoparticles could function similarly to those used in the CDX or PDX models by delivering NIR to abdominal organs via interventional tools, such as optic fiber‐assisted phototherapy.^[^ ^23^ ^]^

### 2.6 Tumor‐Targeting Ability and Safety Assay of ES‐NP_(BTZ; Ce6)_

**Figure 5:**
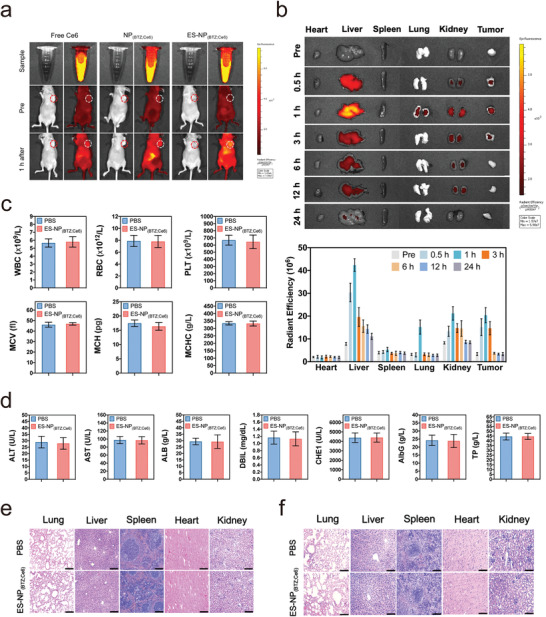
Tumor‐targeting potential and biosafety of ES‐NP_(BTZ; Ce6)_. a) Fluorescence imaging of free Ce6, NP_(BTZ; Ce6)_, and ES‐NP_(BTZ; Ce6)_ at 1 h postintravenous injection. b) Ex vivo fluorescence images of tumors and major organs collected from mice treated with free Ce6 and ES‐NP_(BTZ; Ce6)_ after intravenous injection at different time points (0.5, 1, 3, 6, 12, 24 h) (top) and statistical plot of the radiant efficiency in different groups (bottom). c) Healthy mice were intravenously injected every 3 d for a total of six times with PBS or ES‐NP_(BTZ; Ce6)_ and sacrificed at day 20 for hematological analysis. d) Healthy mice intravenously injected every 3 d for a total of six times with PBS or ES‐NP_(BTZ; Ce6)_ and sacrificed at day 20 for blood biochemical analysis. e) H&E staining analysis of in vivo toxicology for CDX mice model. f) H&E staining analysis of in vivo toxicology for PDX mice model. The data are represented as mean ± SD, *n* = 6 mice per group. Scale bar = 100 µm.

For tumor‐targeting evaluation, Ce6, NP_(BTZ; Ce6),_ and ES‐NP_(BTZ; Ce6)_ were intravenously injected to observe the biodistribution of nanoparticles through in vivo imaging. At 1 h postinjection, compared with Ce6, both ES‐NP_(BTZ; Ce6)_ and NP_(BTZ; Ce6)_ have the fluorescence accumulation at the tumor site, but the fluorescence intensity in ES‐NP_(BTZ; Ce6)_ was much higher than that of NP_(BTZ; Ce6)_, confirming the strong tumor‐targeting and retention ability of ES‐NP_(BTZ; Ce6)_ via ES‐mediated endocytosis (**Figure** **5**a). Notably, more NP_(BTZ; Ce6)_ accumulated around the tumor than free Ce6 due to the transcytosis of nanoparticles.^[^ ^24^ ^]^ For blood circulation assay, the tumors and organs from nude mice (heart, lung, liver, kidney, and spleen) were excised at 0.5, 1, 3, 6, 12, 24 h postinjection of ES‐NP_(BTZ; Ce6)_ through ex vivo imaging (Figure 5b). The fluorescence intensity revealed that ES‐NP_(BTZ; Ce6)_ is mainly distributed in the liver, tumor, and kidneys. Notably, ES‐NP_(BTZ; Ce6)_ was completely metabolized after 24 h. In contrast, most nanoparticles were metabolized at a slower rate, over 48 h after administration.^[^ ^25^ ^]^ This is due to our zeta potential (‐29.1 mV) that grants ES‐NP_(BTZ; Ce6)_ excellent particle dispersion coupled with ER‐mediated quick cell uptake, resulting in rapid metabolism.^[^ ^26^ ^]^

We further evaluated the systemic toxicology of ES‐NP_(BTZ; Ce6)_ on day 20 for biosafety evaluation. Compared with the control group, there was no difference in the hematological markers and liver function in healthy mice (Figure 5c,d; Figure S23, Supporting Information). Analysis based on the H&E staining section of major organs (heart, lung, liver, kidney, and spleen) indicated no clear variation compared to the untreated mice in both CDX and PDX models (Figure 5e,f).

## 3 Conclusion

In this study, we have developed BTZ‐loaded bioresponsive nanoparticles with ES for GBC‐targeted therapy. The unique design of ES‐NP_(BTZ; Ce6)_ provides several merits, including: 1) the simple yet effective self‐assembly synthesis procedures from two polymeric ligands can potentially help expedite the clinical translation of nanomedicine; 2) due to ER on both the cell and nucleus membranes, ES‐NP_(BTZ; Ce6)_ can rapidly enter the cells and accumulate near the nucleus in the tumor cells with relatively high ER expression, which shows strong tumor‐targeting ability both in vivo and in vitro; and 3) under the acidic TME and 808 nm laser irradiation, our ES‐NP_(BTZ; Ce6)_ drug delivery system requires lower BTZ concentrations in combination with moderate ROS generated by Ce6 to destroy the “bounce‐back” response proteins around the nucleus such as DDI2 and p97, which effectively inhibit proteasomes. Thus, the toxicity of BTZ in solid tumors can be substantially decreased.

## Conflict of Interest

Z.G. is a scientific co‐founder of ZenCapsule, Inc. The remaining authors declare no conflict of interest.

## Supporting information

Supporting Information

## Data Availability

The data that support the findings of this study are available from the corresponding author upon reasonable request.
