# Supplementary material for: Bortezomib‐Encapsulated Dual Responsive Copolymeric Nanoparticles for Gallbladder Cancer Targeted Therapy
Source: Adv Sci (Weinh). 2022 Jan 23;9(7):2103895. doi: 10.1002/advs.202103895 (PMC8895115; doi:10.1002/advs.202103895)
Supplement: Supplementary file 1 — Supporting Information [file ADVS-9-2103895-s001.pdf]

ADVANCED SCIENCE
Open Access

Supporting Information

for *Adv. Sci.,* DOI: 10.1002/advs.202103895

Bortezomib-Encapsulated Dual Responsive Copolymeric Nanoparticles for Gallbladder Cancer Targeted Therapy

Mingyu Chen, Sarun Juengpanich, Shijie Li, Win Topatana, Ziyi Lu, Qiang Zheng, Jiasheng Cao, Jiahao Hu, Esther Chan Hui, Lidan Hou, Jiang Chen, Fang Chen, Yu Liu, Sukanda Jiansirisomboon, Zhen Gu, Suparat Tongpeng*, and Xiujun Cai**

Supporting Information

Bortezomib-Encapsulated Dual Responsive Copolymeric Nanoparticles for Gallbladder Cancer Targeted Therapy

Mingyu Chen, Sarun Juengpanich, Shijie Li, Win Topatana, Ziyi Lu, Qiang Zheng, Jiasheng Cao, Jiahao Hu, Esther Chan Hui, Lidan Hou, Jiang Chen, Fang Chen, Yu Liu, Sukanda Jiansirisomboon, Zhen Gu, Suparat Tongpeng*, Xiujun Cai**

M. Chen#, S. Juengpanich#, S. Li#, W. Topatana#, Z. Lu, Q. Zheng, J. Cao, J. Hu, L. Hou, J. Chen, Prof. Z. Gu*, Prof. X. Cai*
Department of General Surgery, Sir Run-Run Shaw Hospital, Zhejiang University, Hangzhou 310016, China
E-mail: srrsh_cxj@zju.edu.cn (X. Cai); guzhen@zju.edu.cn (Z. Gu)

M. Chen#, S. Juengpanich#, S. Li#, W. Topatana#, Prof. X. Cai*
School of Medicine, Zhejiang University, Hangzhou 310058, China

Z. Lu, Prof. Z. Gu*
College of Pharmaceutical Sciences, Zhejiang University, Hangzhou 310058, China

E. Hui
School of Physical and Mathematical Sciences, Nanyang Technological University, Singapore 637371

F. Chen
Department of Chemistry, Zhejiang University, Hangzhou 310027, China

Y. Liu
College of Life Sciences, Zhejiang University, Hangzhou 310058, China

S. Jiansirisomboon, Dr. S. Tongpeng*
School of Ceramic Engineering, Institute of Engineering, Suranaree University of Technology, Nakhon Ratchasima 30000, Thailand
E-mail: tongpengst@gmail.com (S. Tongpeng)

# These authors contributed equally to this work.

Keywords: drug delivery, gallbladder cancer, nanomedicine, proteasome inhibitor, targeted therapy

Table of Contents

Supporting Text Page 3-15

Supporting Figures Pages 16-30

Supporting Text

Materials: All precursors and solvents were obtained commercially and used without further purification. β-benzyl-L-aspartate (BLA), bis-(trichloromethyl)-carbonate (triphosgene), tetrahydrofuran (THF), hexane, estrone, succinic anhydride, dopamine hydrochloride (DA), n-Butylamine, ammonia solution, 1,3-diphenylisobenzofuran (DPBF), acetonitrile, and chlorin e6 (Ce6) were purchased from Aladdin Co. (China). Polyethylene glycol 2000 (PEG_{2K}), N,N-dimethylformamide (DMF), and dimethyl sulfoxide (DMSO) were purchased from Sigma-Aldrich Co. (USA). Bortezomib (BTZ) was purchased from MCE Co. (USA).

Hydrophilic chain synthesis (ES-PEG_{2K}-COOH): To prepare the hydrophilic chain, polyethylene glycol 2000 (PEG_{2K}) (5.0 g, 2.5 mmol) was dissolved in N,N-dimethylformamide (DMF) (30 mL). PEG-COOH was obtained by the reaction of PEG_{2K} with succinic anhydride (0.3 g, 2.5 mmol), and estrone (0.7 g, 2.5 mmol) were then added to the mixture after dissolution as a receptor, respectively. The mixture was stirred and heated in an oil bath to 35°C for 24 h. After 24 h, the reaction mixture was centrifuged to precipitate ES-PEG_{2K} from DMF. ES-PEG_{2K} was purified by precipitation in ethanol (50.0 mL) 3 times. Finally, the precipitate was lyophilized to obtain the hydrophilic chain.

Hydrophobic chain synthesis (n-butylamine-P(Asp-DA)): The hydrophobic chain was synthesized using β-Benzyl-L-aspartate N-carboxy anhydride (BLA-NCA) as a template. BLA-NCA was synthesized by the Fuchs-Farthing method with bis-(trichloromethyl)-carbonate

(triphosgene).[1] Briefly, BLA (10.0g, 44.8 mmol) was suspended in tetrahydrofuran (THF) (100 mL) containing triphosgene (10.0g, 33.7 mmol) and stirred at 60°C for 3 h under argon gas. The reaction mixture was cooled to room temperature and filtered. Next, the product was purified via precipitation with hexane (500 mL) three times, collected by filtration, and dried under vacuum. The resulting BLA-NCA is obtained as a white powder. To prepare the hydrophobic chain, monomer BLA-NCA (10.0 g, 40.0 mmol) was dissolved in DMF (30 mL) and was synthesized by ring-opening polymerization using n-butylamine (0.1 mL) as an initiator. The reaction mixture was stirred and heated in an oil bath to 40°C for 48 h under argon gas. After 48 h, the reaction mixture was cooled to room temperature and the ammonolysis reaction of ammonia solution (1.0 mL) was added to the mixture and stirred at room temperature. The reaction mixture was centrifuged to remove the phenyl group from the solution, and the solution was collected. Catechol functional group was synthesized via dopamine, in which the precursor, dopamine hydrochloride (DA) (0.9 g, 5.0 mmol), was added to the solution and the reaction mixture was stirred and heated in an oil bath to 40°C for 24 h under argon gas. After 24 h, the reaction mixture was cooled to room temperature and cool ethanol was added to precipitate n-butylamine-P(Asp-DA). n-butylamine-P(Asp-DA) was purified by precipitation in cool ethanol (50.0 mL) 3 times. Finally, the precipitate was lyophilized to obtain the hydrophobic chain.

ES-PEG$_{2K}$-P(Asp-DA)-Ce6-BTZ (ES-NP$_{(BTZ; Ce6)}$) synthesis: Briefly, the hydrophobic chain (51.6 mg, 0.0096 mmol) was dissolved in methanol (4.0 mL) as the solvent. A solution of BTZ (3.7 mg, 0.0096 mmol) in methanol (1.0 mL) was added and stirred for 1h at room temperature. P(Asp-DA)-BTZ was prepared by the reaction between the hydroxyl group of dopamine and BTZ. After 1 h, a solution of chlorin e6 (Ce6) (22.8 mg, 0.0096 mmol) in methanol (3.0 mL) was added and stirred for 1 h at room temperature. Subsequently, P(Asp-DA)-Ce6-BTZ was prepared by the reaction between the amino group of the hydrophobic chain and the carboxyl

group of Ce6. A solution of hydrophilic chain (22.6 mg, 0.0096 mmol) in methanol (2.0 mL) was added and stirred for 1 h at room temperature. Finally, methanol was removed via rotary evaporation under vacuum and lyophilized to obtain the pH-responsive polymer. The polymeric nanoparticles were re-dispersed in PBS for subsequent studies.

Characterization: Transmission electron microscopy (TEM) was taken with HT-7700 (Hitachi, Japan) at 200 kV for morphology characterization. The samples were prepared by a small drop (0.1 mg/mL) of sample solution on a carbon-coated copper grid and were dried before TEM observation. Zeta potentials and nanoparticle size were investigated using dynamic light scattering (DLS) on Zetasizer Nano ZS90 (Malvern Instruments, UK). For zeta potential and size measurement, a standard electrophoresis mini-cell was used. Before measurement, the samples (0.1 mg/mL) were dissolved in PBS and subjected to ultrasound sonication for 10 min. The data from 100 measurements repeated with 3 cycles were collected on the auto-correlator with detection angle of scattered light and were averaged to obtain the mean standard deviation (SD). ^{1}H-NMR analysis was conducted on Ascend 600 (Bruker, Swiss) using DMSO-d_6 as the solvent. Nanoparticles (6 mg) were reacted with 0.6 mL DMSO-d_6 during ^{1}H-NMR measurement. FTIR analysis was conducted on Nicolet AVA TAR370 (ThermoFisher, USA). The spectral were recorded with a resolution of 2 cm-1 and the powder samples were compressed by KBr pellets. Raman spectroscopy analysis was conducted on InVia Reflex Raman Microscope (Renishaw, UK) with an excitation wavelength of 532 nm. HPLC analysis was conducted on Agilent 1100 (Marshall Scientific, USA). Absorption spectrum was conducted on Agilent Cary 6 (Marshall Scientific, USA). Fluorescence spectrum was conducted on Cary Eclipse (Agilent, USA) with various excitation wavelengths. NIR light source was supplied by an optical fiber-coupled 808 nm diode laser (STL808T1-7W, Radium Laser Co., China).

ES-NP$_{(BTZ; Ce6)}$ transmittance measurement at different pH values: The light-transmittance of ES-NP$_{(BTZ; Ce6)}$ solutions (0.1 mg/mL) was measured using UV-vis spectrophotometer at 502 nm with a gradual decrease of the solution pH value from 7.5 to 4.0 by adding 0.1 M H_2SO_4.

Fluorescence intensity measurement at different pH values: The fluorescence intensity of ES-NP$_{(BTZ; Ce6)}$ solutions (0.1 mg/mL) was measured using a fluorescence spectrophotometer at 400 nm excitation with solution pH adjustments from 7.4 to 5.5 by adding 0.1 M H_2SO_4.

Singlet oxygen generation measurement at different pH values: DPBF mixed with acetonitrile (20 μL, 8 mM) was added into ES-NP$_{(BTZ; Ce6)}$ solutions (0.1 mg/mL, 3 mL) at different pH and was irradiated with 808nm laser source (2.0 W cm^{-2}) according to the designated time intervals (0 min, 5 min, 10 min, 20 min, and 30 min). The absorption spectra were then measured after the designated time intervals of NIR irradiation using a UV-vis spectrophotometer. All samples were thoroughly mixed and stirred during laser irradiation to ensure the dissipation of light energy throughout the sample. All experiments were performed in triplicate.

In vitro drug release: 3 mL of fresh PBS was placed in an MWCO 1 kDa dialysis tube (Spectra/Por, USA). The dialysis tube was then immersed into ES-NP$_{(BTZ; Ce6)}$ PBS solutions (1 mg, 10 mL) at different pH values under stirring at 100 rpm, 37°C. At the designated time intervals (0 h, 1 h, 3 h, 5 h, 7 h, 12 h, 24 h, 36 h, 48 h, 60 h, and 72 h), all 3 mL of PBS was removed from the dialysis tube and replaced with fresh PBS. The amount of BTZ released was measured using a UV-vis spectrophotometer at 270 nm. For samples with NIR irradiation, the samples were thoroughly mixed and stirred during laser irradiation with an 808 nm laser source at a power density of 2.0 W cm^{-2} for 5 min to ensure the dissipation of light energy throughout the sample. All experiments were performed in triplicate.

Cell culture: Human gallbladder carcinoma cell line GBC-SD, human cholangiocarcinoma cell line RBE, HuCCT1, and CCLP1 were originally purchased from Shanghai Institute for Biological Science, Chinese Academy of Science (Shanghai, China); Human gallbladder carcinoma cell line NOZ, EH-GB1, SGC-996, and human intrahepatic biliary epithelial cell line HIBEC were provided by Prof. Ying-Bin Liu's lab at Xinhua Hospital Affiliated to Shanghai Jiao Tong University School of Medicine, China. GBC-SD, RBE, HuCCT1, CCLP1, NOZ, and EH-GB1 were cultured in Dulbecco's Modified Eagle Medium (DMEM) supplemented with 10% fetal bovine serum (FBS) and 1% penicillin/streptomycin at 37°C in 5% CO_2. SGC-996 and HIBEC were cultured in Roswell Park Memorial Institute (RPMI) 1640 supplemented with 10% fetal bovine serum (FBS) and 1% penicillin/streptomycin at 37°C in 5% CO_2.

Cellular uptake: GBC-SD and NOZ cells were exposed to ES-NP$_{(BTZ;\ Ce6)}$ at tumorous microenvironment pH 6.5 and physiological pH 7.4 to verify cellular uptake. GBC-SD cells (5 × 10^4/mL) and NOZ cells (5 × 10^4/mL) were seeded in 1mL medium in confocal dishes and incubated overnight and then ES-NP$_{(BTZ;\ Ce6)}$ (0.5 μg/mL) was added to the confocal dishes. After incubating for 4 h, 12 h, and 24 h, cells were washed with PBS three times. The samples were fixed with 4% paraformaldehyde for 10 min and incubated with DAPI (0.01%) for 10 min. Cellular uptake was monitored by confocal microscopy (Zeiss LSM 880, Germany) at different time points.

Cyro-TEM cell imaging: GBC-SD and NOZ cells were exposed to ES-NP$_{(BTZ;\ Ce6)}$ at tumorous microenvironment pH 6.5 to verify cellular uptake. GBC-SD cells (2 × 10^6/mL) and NOZ cells (2 × 10^6/mL) were seeded in 6 cm Petri dishes until adherent and replaced with ES-NP(BTZ; Ce6) (0.5 μg/mL). After incubation for 4 h and 24 h, the cells were first fixed with 2.5% glutaraldehyde in PBS (0.1 M, pH 7.0) overnight, washed three times with PBS, and subsequently post-fixed with 1% OsO_4 in PBS for 1h and washed three times with PBS. Next,

the cell samples were dehydrated by a graded series of ethanol (30%, 50%, 70%, 80%) and acetone (90%, 95%, 100%) for 15 min. The cell samples were then placed in a mixture of 1:1 and 1:3 acetone and Spurr resin mixture for 1 h and 3 h respectively, and transferred to the final Spurr resin mixture overnight. Finally, the specimens were placed in Spurr resin and heated to 70°C for 9 h, and were sectioned into ultrathin slices with LEICA EM UC7. The sections were then stained with uranyl acetate and alkaline lead citrate for 10 min. The morphology was observed using TEM microscopy TecnaiG2 Spirit (Thermo Fisher Scientific, USA) at 120kV.

Cell Counting Kit-8 (CCK-8) Assay: The in vitro cytotoxicity of nanoparticles was measured using the CCK-8 (Yeasen, Shanghai) assay according to the manufacturer's instructions. GBC-SD, NOZ, and HIBEC cells were seeded into 96-well cell culture plates at 0.6×10^4 per well until adherent and replaced with BTZ in different concentrations (3.16 – 316 nM) or ES-NP$_{(BTZ; Ce6)}$ (3.16 – 316 nM, BTZ concentration in ES-NP$_{(BTZ; Ce6)}$) and cultured for 48 h. The absorbance was measured by a microplate reader (Thermo Fisher Scientific, USA) at 450 nm. All experiments were performed in triplicate.

Cell damage caused by ROS production from Ce6 in nanoparticles under laser irradiation was measured in the same manner. GBC-SD and NOZ cells were seeded into 96-well cell culture plates at 0.6×10^4 per well until adherent and replaced with ES-NP$_{(Ce6)}$ loaded with different concentrations of Ce6 (0.07 – 6.73 μg/mL, Ce6 concentration in ES-NP$_{(Ce6)}$) for 24 h incubation. After removal of nanoparticles, cells were transferred into fresh media and irradiated by the 808 nm laser source at a power density of 2.0 W cm^{-2} for 5 min with a 1 min interval. The cells were then incubated at 37°C for an additional 24 h before measuring on a microplate reader at 450 nm to determine their viabilities relative to the control unirradiated cells. All experiments were performed in triplicate.

Likewise, the effect of nanoparticles on GBC cell proliferation was also measured by CCK-8 assay nanoparticles cell proliferation assay. GBC-SD and NOZ cells were seeded in 96-well plates at 0.6×10^4 per well until adherent and replaced with different media for 24 h incubation. Briefly, each cell line was divided into 8 groups: (1) NC (normal media), (2) NC (normal media) with laser irradiation, (3) BTZ (10 nM for GBC-SD, 5 nM for NOZ), (4) BTZ (10 nM for GBC-SD, 5 nM for NOZ) with laser irradiation, (5) ES-$NP_{(Ce6)}$ (same nanoparticle concentration as group 7), (6) ES-$NP_{(Ce6)}$ (the same nanoparticle concentration as group 7) with laser irradiation, (7) ES-$NP_{(BTZ;\ Ce6)}$ (10nM, BTZ concentration in ES-$NP_{(BTZ;\ Ce6)}$ for GBC-SD; 5 nM, BTZ concentration in ES-$NP_{(BTZ;\ Ce6)}$ for NOZ), and (8) ES-$NP_{(BTZ;\ Ce6)}$ (10nM, BTZ concentration in ES-$NP_{(BTZ;\ Ce6)}$ for GBC-SD; 5 nM, BTZ concentration in ES-$NP_{(BTZ;\ Ce6)}$ for NOZ) with laser irradiation. After removal of old media, cells were transferred into fresh media, and groups 2, 4, 6, and 8 were irradiated by an 808 nm laser source at a power density of 2.0 W cm^{-2} for 5 min with 1 min interval. The cells were then incubated at 37°C for an additional 24 h before measuring on a microplate reader at 450 nm to determine their viabilities relative to the control unirradiated cells. All experiments were performed in triplicate.

Furthermore, the effect of nanoparticles on other cancer cell proliferation was verified in the same methods mentioned previously. Each cancer cell line was divided into 3 groups: (1) NC (normal media), (2) BTZ (10 nM), and (3) ES-$NP_{(BTZ;\ Ce6)}$ (10 nM, BTZ concentration in ES-$NP_{(BTZ;\ Ce6)}$) with laser irradiation. For PANC-1, more concentrations 31.6, 100, and 316 nM (in terms of BTZ dose) were investigated in the same way.

Western Blotting: For detection of estrogen receptors, HIBEC, NOZ, GBC-SD, EH-GB1, SGC-996, RBE, HuccT1, and CCLP1 cells were seeded in 6-well plates at the density of 3×10^5 per well. After 48 h, the total protein of each cell line was extracted respectively. Major healthy organs heart, liver, spleen, lung, kidney, and gallbladder tumors originating from tumor-bearing BALB/c nude mice were also extracted. For validation of mechanism, GBC-SD

and NOZ cells were seeded in 6-well plates at the density of 3×10^5 per well until adherent and replaced with different media. Each cell line was also divided into 8 groups and treated like the cell proliferation assay mentioned previously. After 48 h, total protein was extracted from each cell group. Then western blotting was performed according to the standard methods. The following antibodies, anti-ubiquitin (sc-166553) and anti-DDI2 (sc-514004) were obtained from Santa Cruz Biotechnology, and anti-estrogen receptor alpha (ab108398), anti-estrogen receptor beta (ab288), anti-p97 (ab109240), anti-Nrf1 (ab175932), anti-GAPDH (ab8245), and anti-Lamin B1 (ab16048) were obtained from Abcam.

PI/Annexin V apoptosis assay: Briefly, GBC-SD and NOZ cells were seeded in 24-well plates at 1×10^5 per well until adherent and replaced with different media for 24 h incubation. Each cell line was also divided into 8 groups and treated similarly as mentioned previously. After 48 h, all cells in each group including both floating and attached cells were collected by trypsinization (0.25% Trypsin, without EDTA (Gibco) and washed with PBS. The apoptotic cells were detected by Annexin V-FITC Apoptosis Detection Kit I (BD Biosciences) by staining with Annexin V-FITC and PI according to the supplier's instructions. Viable and dead cells were detected by a flow cytometer (BD LSRFortessa™, USA).

Wound healing assays: Briefly, GBC-SD and NOZ cells were seeded in ibidi Culture-Insert (ibidi GmbH, Martinsried, Germany) at 5×10^4 per Culture-Insert on 12-well plates. After appropriate cell attachment, the Culture-Insert was gently removed, and photomicrographs were taken under a microscope (Zeiss, Germany) at 0 h. Next, each cell line was divided into 8 groups and treated as cell proliferation assay mentioned previously. After wounding for 48 h, each wound was captured by a microscope (Zeiss, Germany). All experiments were performed in triplicate.

Colony-forming assays: Briefly, GBC-SD and NOZ cells were seeded in 24-well plates at 1 $\times 10^4$ per well until adherent and replaced with different media for 24 h incubation. Each cell line was also divided into 8 groups and treated similarly as mentioned previously. Then, all 8 groups were seeded in 6-well plates at 1 $\times 10^3$ per well. Next, each group was cultured for an additional week. Finally, each well was fixed with 4% paraformaldehyde and stained with 0.1% crystal violet according to the manufacturer's instructions, and a full visual was captured by a gel imager (Bio-Rad, USA). All experiments were performed in triplicate.

ROS assays: Briefly, GBC-SD and NOZ cells were seeded in 24-well plates at 1 $\times 10^5$ per well until adherent and replaced with different media for 24 h incubation. Each cell line was also divided into 8 groups and treated similarly as mentioned previously. 4 hours after irradiation, for flow cytometry analysis, adherent cells of all groups were harvested and washed by PBS 3 times; for observation of the fluorescence cell imager, adherent cells of all groups were washed by PBS 3 times. Next, each group was stained with 500 μL (25 μM) 2 ',7 '-dichlorodihydrofluorescein diacetate (Sigma-Aldrich, USA) for 30 min at 37°C in the dark. The cells were then immediately analyzed by flow cytometry (BD LSRFortessa^{TM}, USA) and observed by a fluorescence cell imager (Bio-Rad, USA), respectively. All experiments were performed in triplicate.

Cell-derived xenograft (CDX) animal model and in vivo treatment: Female BALB/c nude mice (4 weeks old) were obtained from Shanghai SLAC Laboratory Animal Co. and used under protocols approved by the Ethics Committee of Sir Run-Run Shaw Hospital. To generate the NOZ GBC model, 5 $\times 10^6$ NOZ cells in 100 μL PBS were subcutaneously injected into the right armpit of each female BALB/c nude mouse. Mice were treated when the tumor volume approached 80 mm^3. For treatment, mice were divided randomly into 8 groups (6 mice in each group): (1) PBS, (2) PBS with laser irradiation, (3) BTZ (0.25 mg/kg, equivalent to the dose loaded in ES-$NP_{(BTZ;\ Ce6)}$), (4) BTZ (0.25 mg/kg, equivalent to the dose loaded in ES-$NP_{(BTZ;}$

Ce6)) with laser irradiation, (5) ES-NP$_{(Ce6)}$ (10 mg/kg), (6) ES-NP$_{(Ce6)}$ (10 mg/kg) with laser irradiation, (7) ES-NP$_{(BTZ;\ Ce6)}$ (10 mg/kg), and (8) ES-NP$_{(BTZ;\ Ce6)}$ (10 mg/kg) with laser irradiation. The dosage of BTZ is within the range of clinically attainable levels. The mice were intravenously injected with 100 μL of the respective formulations every three days and groups 2, 4, 6, and 8 were irradiated at 1 h post-injection at a power density of 2.5 W cm^{-2} for 10 min, with a 1 min interval after each minute of irradiation to avoid tissue heating. The tumor volume and mouse body weight were measured every two days. Tumor diameters were measured with a digital caliper and tumor volume was calculated using the formula: tumor volume (mm^3) = (tumor length) × (tumor width)^2 / 2. After 20 days, all the mice were euthanized, and tumors were removed from the mice under different treatments, fixed in 4% paraformaldehyde, and embedded in paraffin. Paraffin-embedded tissue slices then underwent H&E and Ki-67-antigen.

In vivo imaging: Free Ce6, NP$_{(BTZ;\ Ce6)}$, ES-NP$_{(BTZ;\ Ce6)}$ were administered by intravenous injection separately for three mice bearing ~80 mm^3 tumors. Fluorescence images of the mice were acquired by the NIR imaging system (PerkinElmer IVIS® Lumina LT, USA) at 1 h post-injection.

Bio-safety assessment: For histopathological analyses of major organs, different groups of mice were sacrificed after the 20 days treatment to collect the major organs (heart, liver, spleen, lung, and kidney). The tissue samples were fixed in 4% paraformaldehyde solution and stained with hematoxylin and eosin and examined under a digital microscope. For blood analysis, healthy mice were intravenously injected every 3 days for a total of 6 times with 100 μL of ES-NP$_{(BTZ;\ Ce6)}$ solution (10 mg/kg) and sacrificed at day 20, and healthy mice with PBS injection were used as the control group. Subsequently, complete blood investigation and serum biochemistry assays were carried out by collecting 600 μL of blood from the mice. The white blood cells (WBC), red blood cells (RBC), mean corpuscular volume (MCV), platelets (PLT), mean corpuscular hemoglobin (MCH), and hemoglobin concentration (MCHC) were measured.

Blood biochemical examination parameters, including alanine aminotransferase (ALT), aspartate aminotransferase (AST), albumin (ALB), direct bilirubin (DBIL), cholinesterase (CHE1), serum albumin (AlbG), and total protein (TP). For blood circulation assay, PBS and ES-NP$_{(BTZ; Ce6)}$ were administered by intravenous injection separately for healthy mice. Next, the mice were sacrificed at different time points (0.5, 1, 3, 6, 12, 24 h) after intravenous injection, with the heart, liver, spleen, lung, and kidney excised for the observation of the biodistribution of nanoparticles via imaging.

Patient-derived xenograft (PDX) animal model and in vivo treatment: We obtained fresh tumor biopsies from GBC patients (according to the protocols approved by the Ethics Committee of Sir Run-Run Shaw Hospital). Upon GBC resection, fresh 2-mm tumor pieces were obtained and transported on ice to the animal surgery suite for subcutaneous implantation into female BALB/c nude mice (4 weeks old). The mice were housed, maintained, and treated under protocols approved by the Ethics Committee of Sir Run-Run Shaw Hospital. Incisions of 1 cm were made on the right armpit of anesthetized female BALB/c nude mice, and blunt dissection of the subcutaneous layer was performed. A viable tumor piece was placed in the armpit flank subcutaneous tissue, and the skin was closed with surgical clips (generation 1). Once engrafted and tumors reached the endpoint (1.5 cm in diameter), tumors were divided evenly into 2 mm pieces and re-implanted female BALB/c nude mice as mentioned previously (generation 2). Generation 3 was generated in the same manner. When the tumors from early-passage (generation 2 or 3) mice reached approximately 80 mm^3, the animals were randomized into two groups (6 mice in each group): (1) PBS, and (2) ES-NP$_{(BTZ; Ce6)}$ (10 mg/kg) with laser irradiation. The subsequent treatments were the same as the CDX model to verify the in vivo effect.

Potential clinical applications and limitations

For deep-located abdominal tumors, our nanoparticles could also function similarly to those used in CDX or PDX models by delivering NIR to abdominal organs via interventional tools, such as optic fiber-assisted phototherapy.[2] Several studies have recently explored the application of interventional therapy-assisted phototherapy in deep in-situ tumors with encouraging results.[2, 3] The same technique may also be applied to treat patients with GBC using currently available laparoscopic equipment. Additionally, our nanoparticles have practical applications based on the clinical characteristics of resected GBC. For instance, positive surgical margins for GBC are frequently associated with poor patient survival.[4] Hence, our nanoparticles combined with NIR irradiation of the margin can significantly help lower the positive rate of the margin during the surgical resection of GBC. In addition, GBC patients who underwent laparoscopic surgical treatment may develop a tumor on the surface of the body as a result of the incision implantation tumor.[5] Furthermore, skin permeability occurs between 620-850 nm, and higher wavelengths have stronger tissue penetration.[6] Thus, employing an 808 nm laser may be more advantageous in practice. Meanwhile, our results indicate that ROS levels generated by Ce6 under 808 nm laser irradiation maintained the effect of combination therapy. Naturally, it is undeniable that current researches on nanomaterials combined with NIR irradiation are usually verified on the surface of animals. Therefore, the treatment of deep tumors in the body with such materials requires further development and validation.

Statistical analysis:

The data in this study were expressed as mean ± SD of at least three independent experiments. OriginPro 9.5.1 and GraphPad Prism 8 Software were used for the statistical analysis. One-way analysis of variance (ANOVA) with Tukey's test was used to compare CCK-8 assay, ROS intensity, wound healing studies, colony-forming assays, tumor volume, body weight, etc., with different treatments. In all cases, the differences of statistics were considered at $*P < 0.05$, $**P < 0.01$. All P values were two-tailed.

References

[1] R. Wilder, S. Mobashery, *The Journal of Organic Chemistry* **1992**, *57*, 2755; D. Ling, H. Xia, W. Park, M. J. Hackett, C. Song, K. Na, K. M. Hui, T. Hyeon, *ACS Nano* **2014**, *8*, 8027.

[2] Y. Hu, C. Chi, S. Wang, L. Wang, P. Liang, F. Liu, W. Shang, W. Wang, F. Zhang, S. Li, H. Shen, X. Yu, H. Liu, J. Tian, *Adv Mater* **2017**, *29*.

[3] A. K. Parchur, G. Sharma, J. M. Jagtap, V. R. Gogineni, P. S. LaViolette, M. J. Flister, S. B. White, A. Joshi, *ACS Nano* **2018**, *12*, 6597; F. Zhang, X. Han, Y. Hu, S. Wang, S. Liu, X. Pan, H. Wang, J. Ma, W. Wang, S. Li, Q. Wu, H. Shen, X. Yu, Q. Yuan, H. Liu, *Adv Sci (Weinh)* **2019**, *6*, 1801507; M. Wang, Y. Li, M. Wang, K. Liu, A. R. Hoover, M. Li, R. A. Towner, P. Mukherjee, F. Zhou, J. Qu, W. R. Chen, *Acta Biomater* **2021**.

[4] C. G. Ethun, L. M. Postlewait, N. Le, T. M. Pawlik, S. Buettner, G. Poultsides, T. Tran, K. Idrees, C. A. Isom, R. C. Fields, L. X. Jin, S. M. Weber, A. Salem, R. C. G. Martin, C. Scoggins, P. Shen, H. D. Mogal, C. Schmidt, E. Beal, I. Hatzaras, R. Shenoy, D. A. Kooby, S. K. Maithel, *JAMA surgery* **2017**, *152*, 143.

[5] A. Prasad, C. Avery, R. J. Foley, *The British journal of surgery* **1994**, *81*, 1697.

[6] K. Szaciłowski, W. Macyk, A. Drzewiecka-Matuszek, M. Brindell, G. Stochel, *Chem Rev* **2005**, *105*, 2647; I. Yoon, J. Z. Li, Y. K. Shim, *Clin Endosc* **2013**, *46*, 7.

Supporting Figures

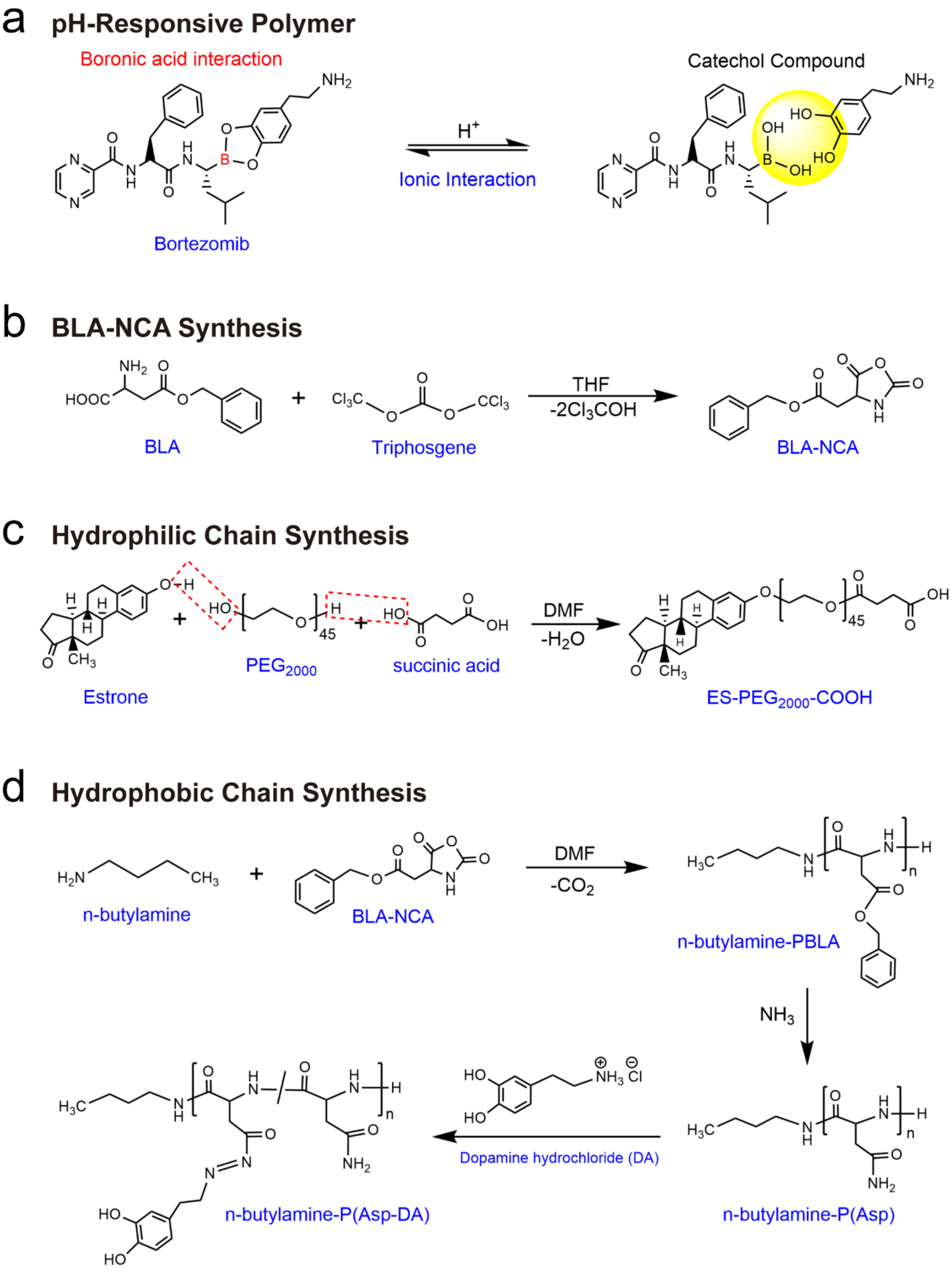

e **Prodrug**

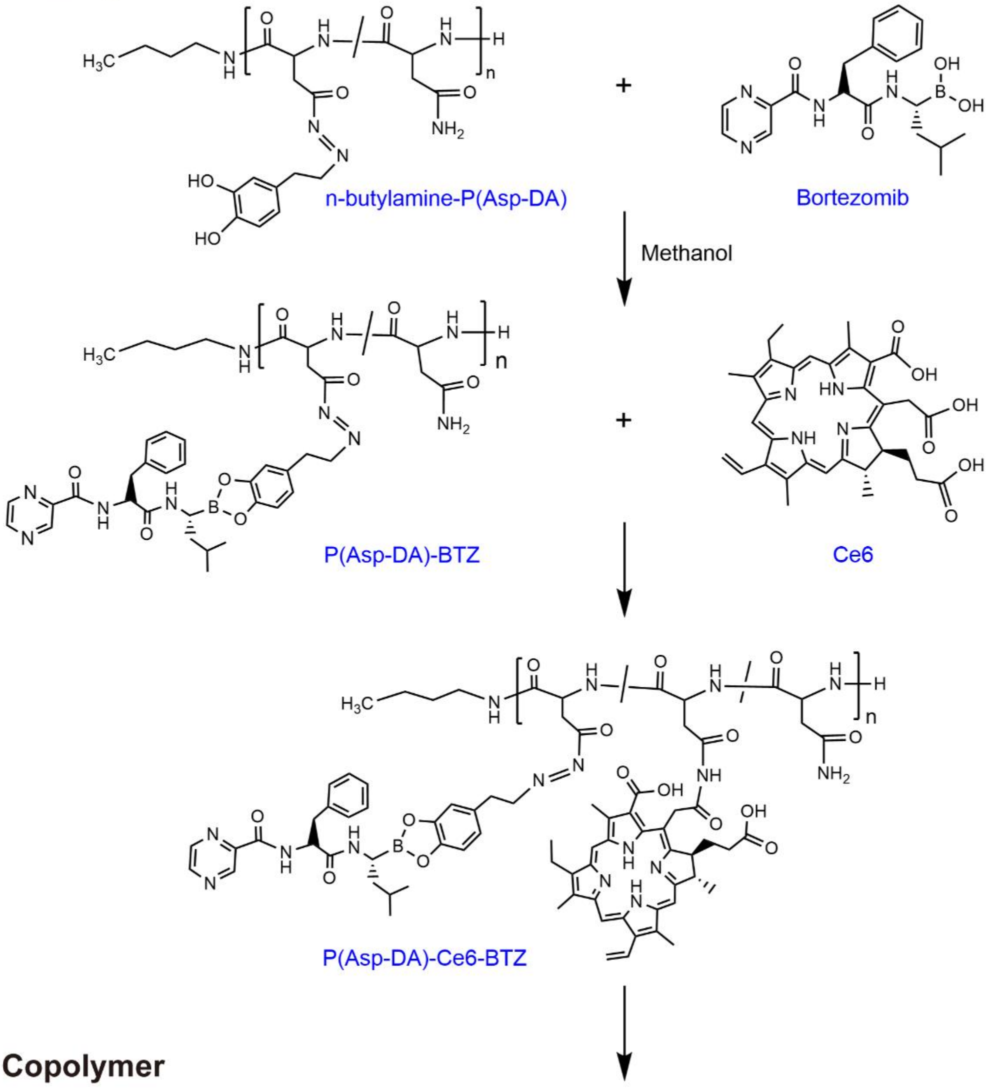

f **Copolymer**

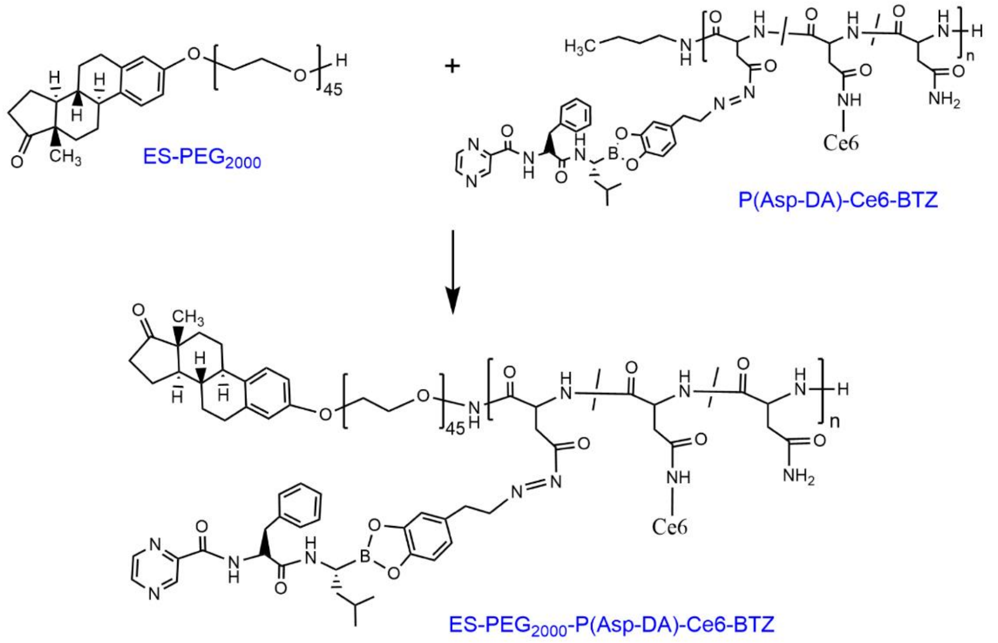

Figure S1: Synthesis scheme of ES-NP$_{(BTZ;\ Ce6)}$ (n=40).

a

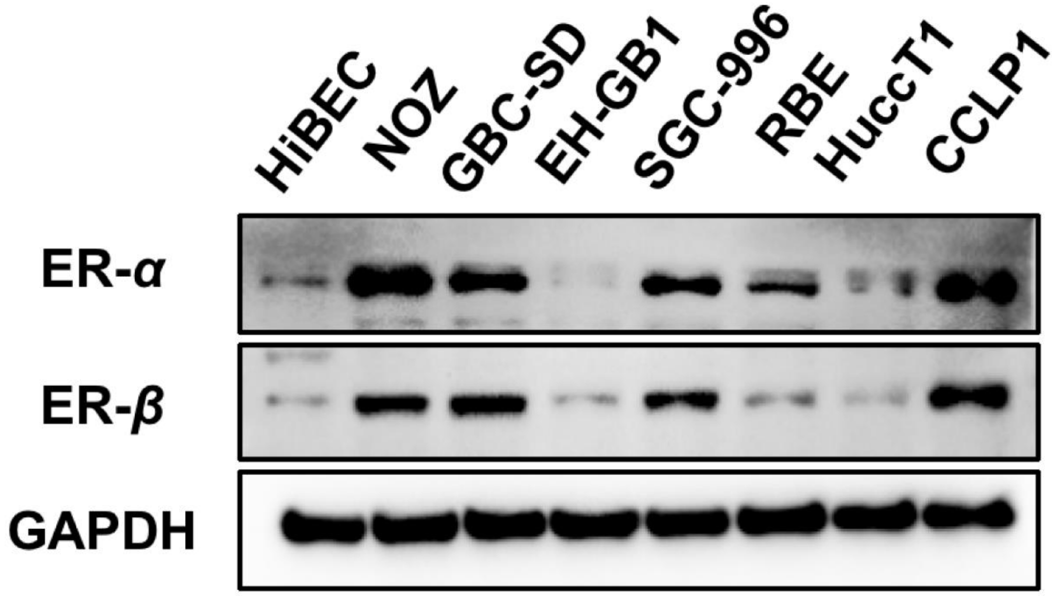

b

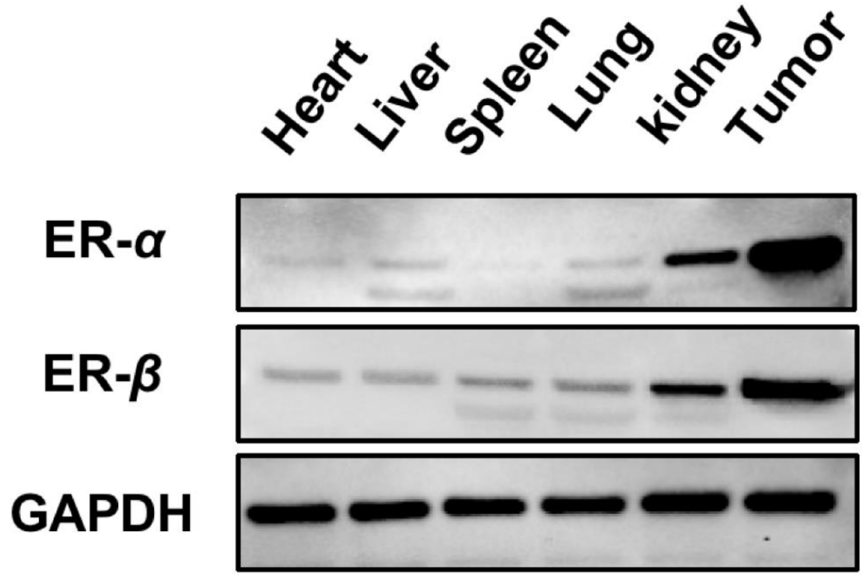

Figure S2: Western blot for estrogen receptor (ER). a) α/β of normal biliary tract tissue and biliary tract tumor cell lines. b) α/β of major healthy organs and gallbladder tumors originating from tumor-bearing BALB/c nude mice.

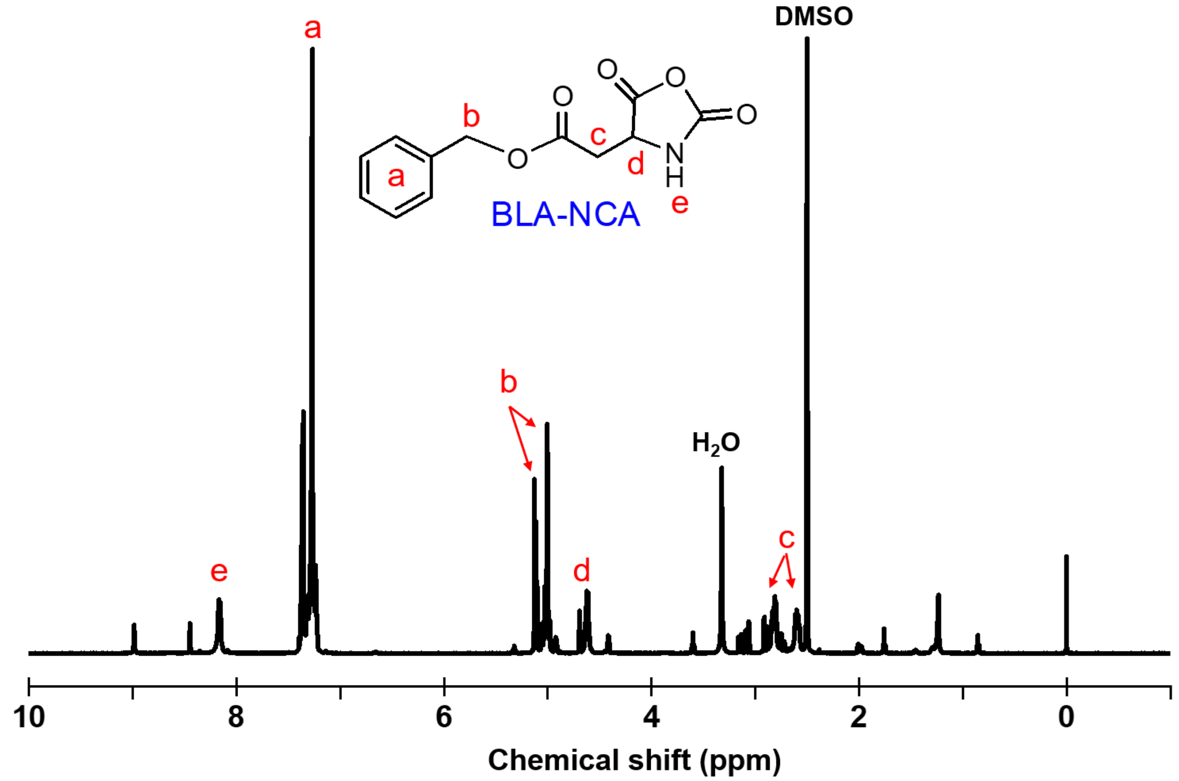

Figure S3: ^{1}H-NMR analysis of BLA-NCA.

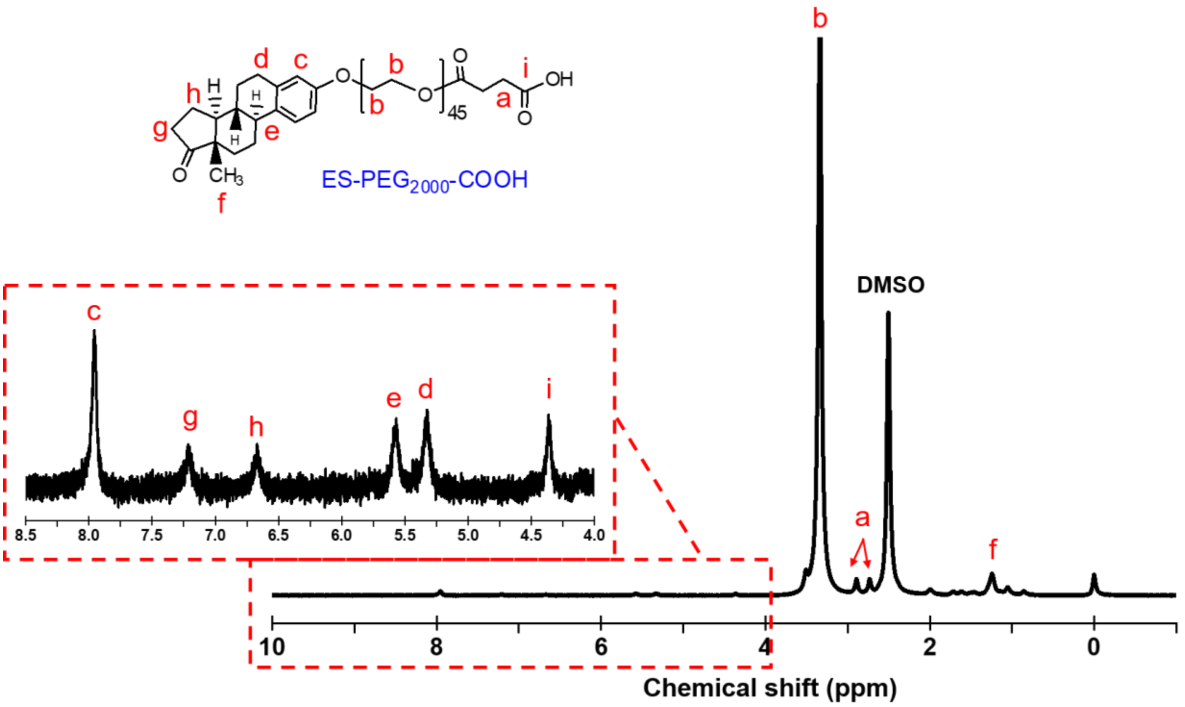

Figure S4: ^{1}H-NMR analysis of hydrophilic chain (ES-PEG$_{2K}$-COOH).

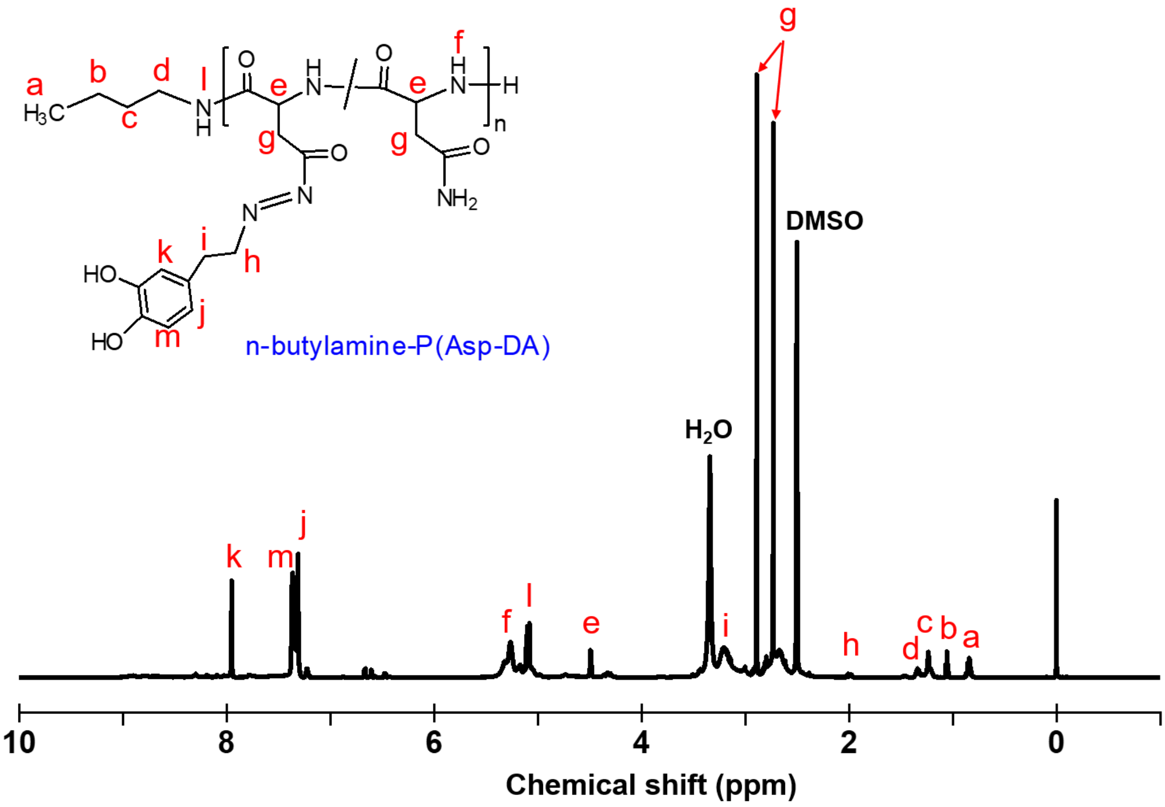

Figure S5: ^{1}H-NMR analysis of hydrophobic chain (n-butylamine-P(Asp-DA)).

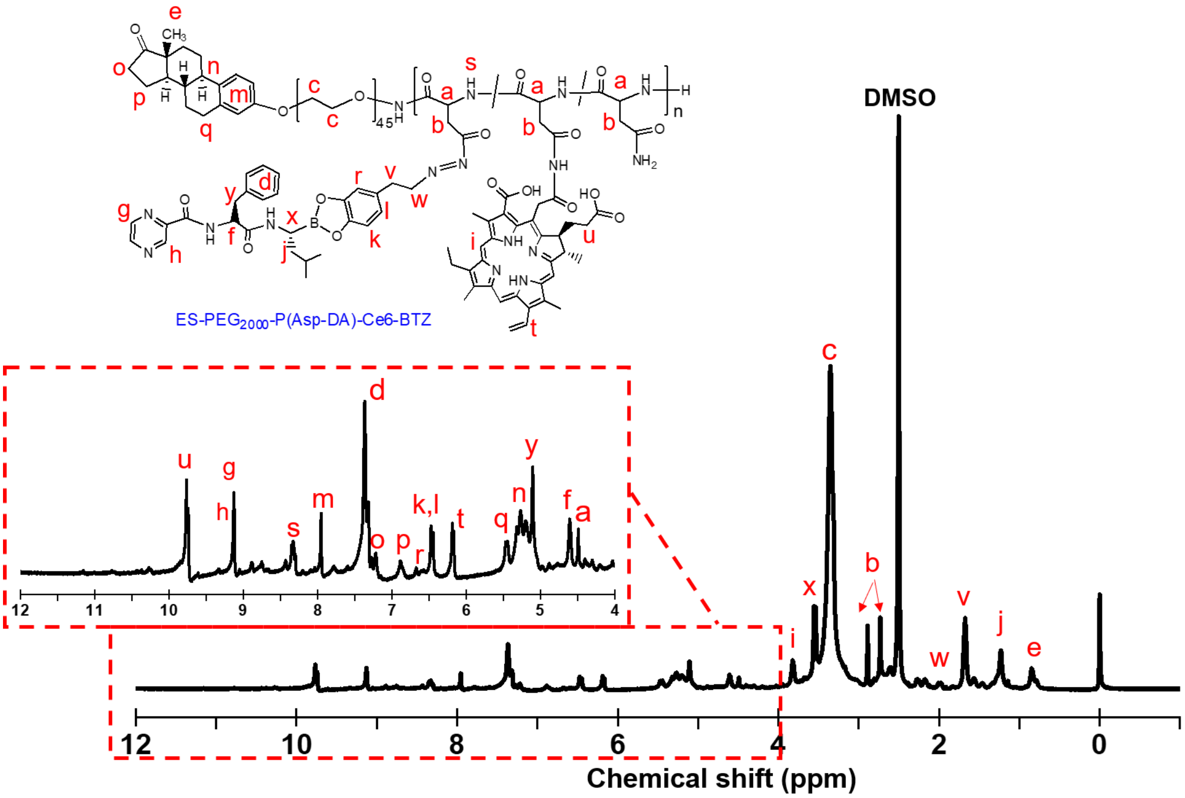

Figure S6: ^{1}H-NMR analysis ES-PEG$_{2K}$-P(ASP-DA)-Ce6-BTZ (ES-NP$_{(BTZ;\ Ce6)}$).

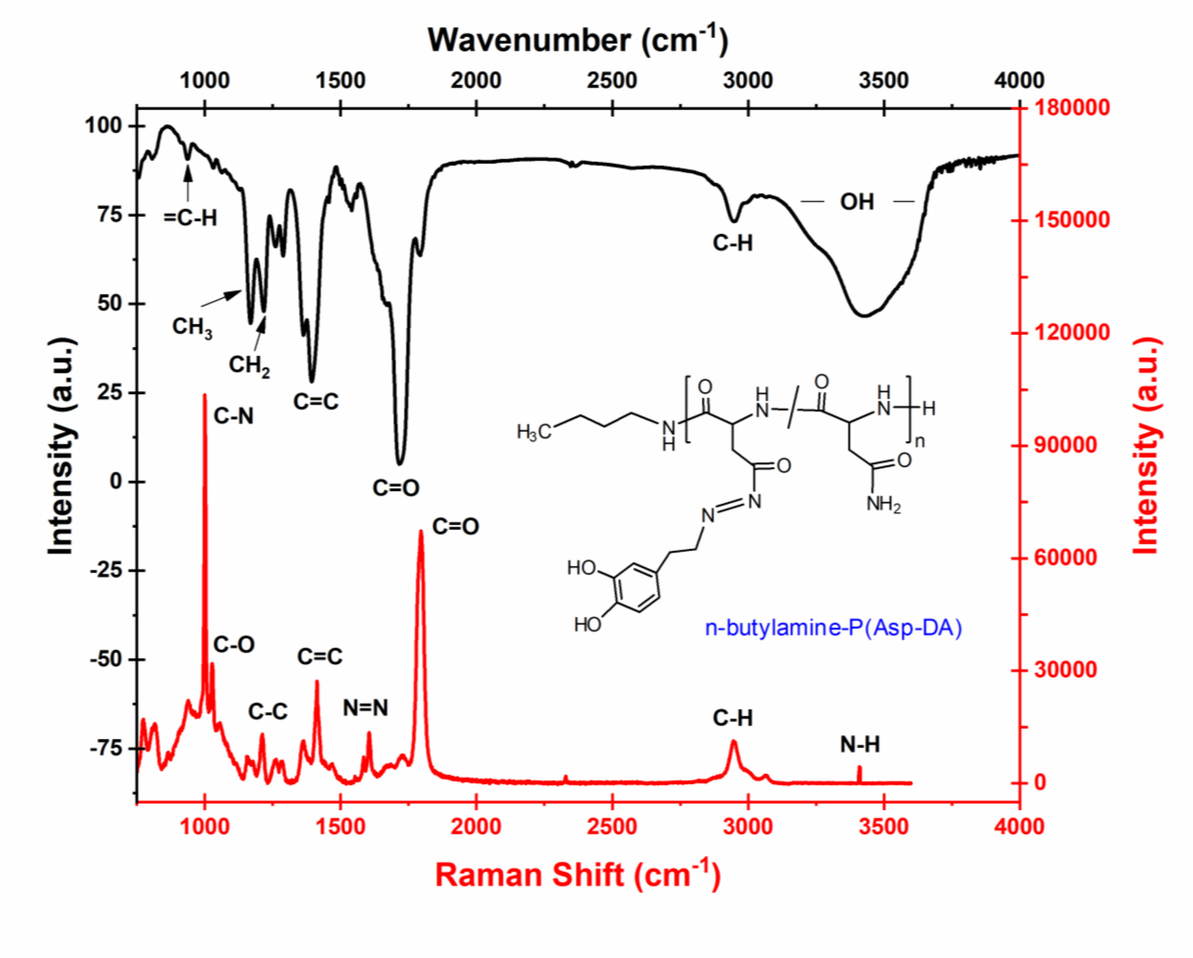

Figure S7: FTIR (black) and Raman (red) spectrometry analysis of hydrophobic chain.

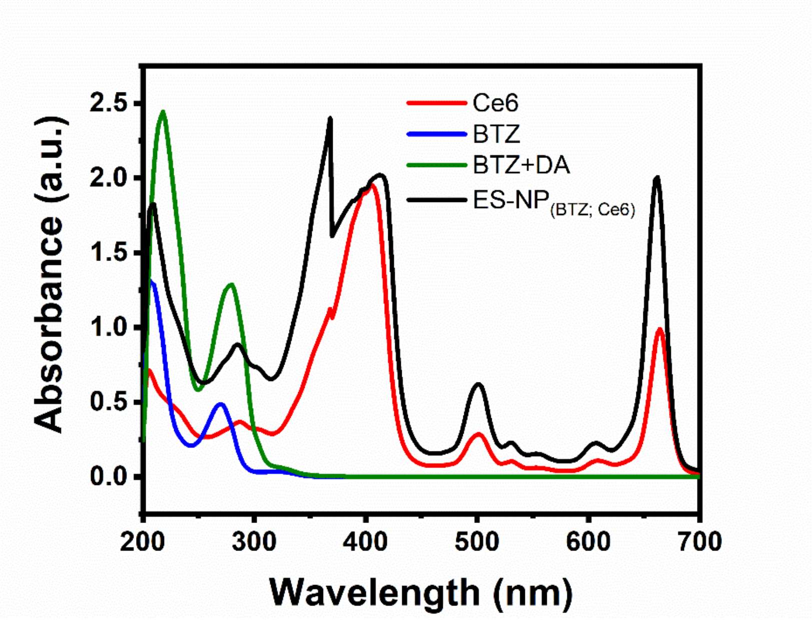

Figure S8: UV-vis spectra of Ce6, BTZ, BTZ-DA, and ES-NP$_{(BTZ;\ Ce6)}$.

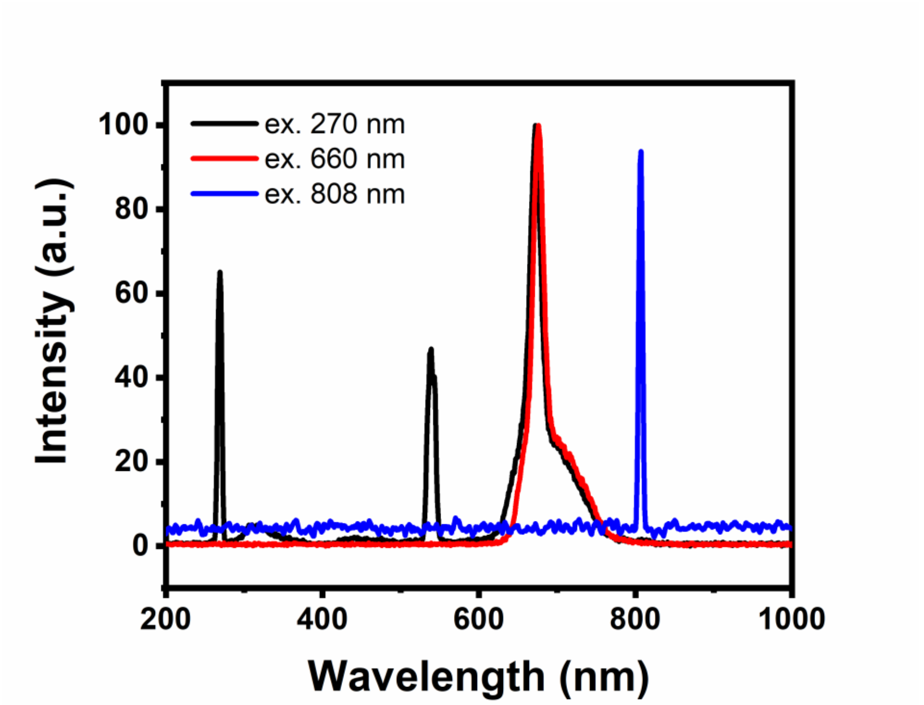

Figure S9: Fluorescence spectra of ES-NP$_{(BTZ;\ Ce6)}$ at different excitation wavelengths.

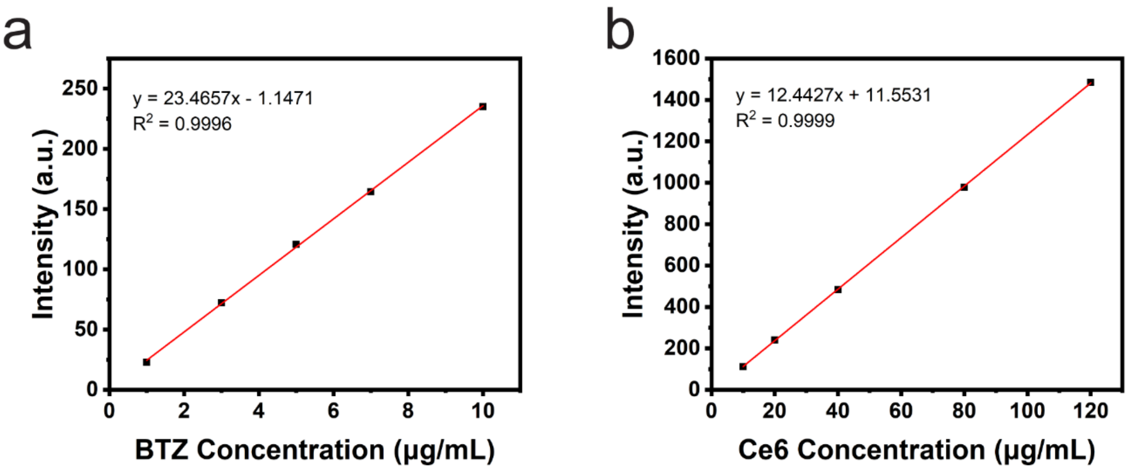

Figure S10: HPLC calibration curve to calculate a) BTZ and b) Ce6 concentration in ES-NP$_{(BTZ;\ Ce6)}$.

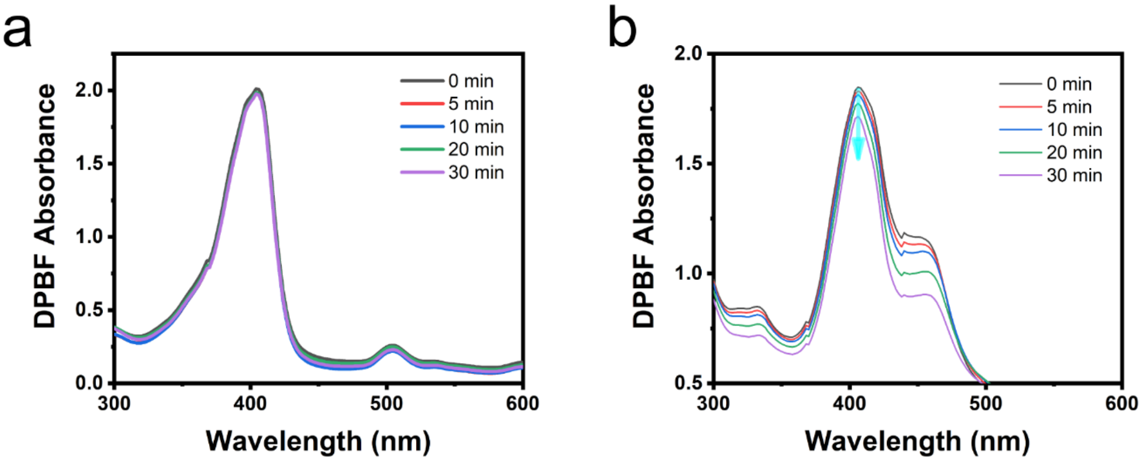

Figure S11: ES-NP$_{(BTZ;\ Ce6)}$ changes in DPBF-absorbance spectrum at a) pH 7.4 and b) pH 6.5 under 808 nm laser irradiation, demonstrating a significant decrease at pH 6.5 and negligible decrease at pH 7.4.

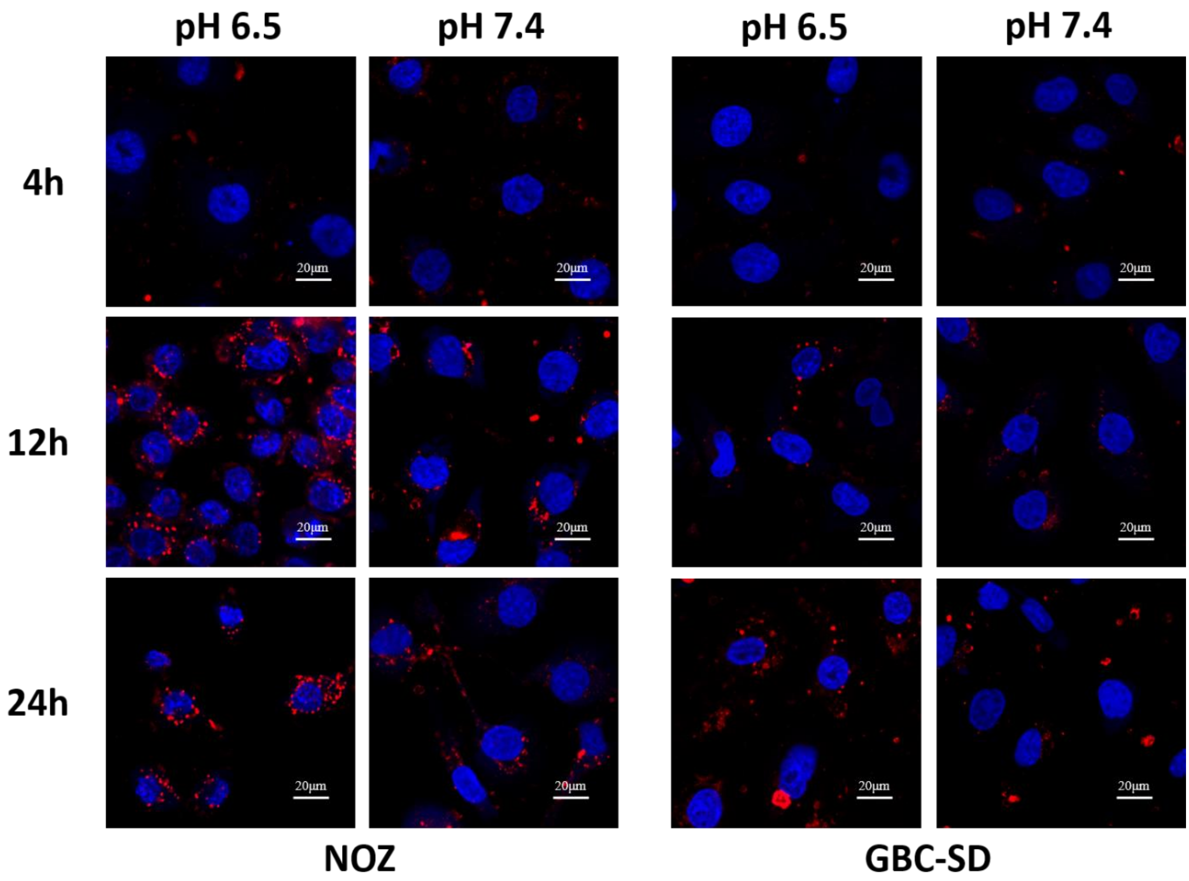

Figure S12: Confocal laser scanning microscope images to demonstrate the pH-dependent cellular uptake of ES-NP$_{(BTZ;\ Ce6)}$. DAPI-stained cells are shown in blue; fluorescence signals from Ce6 in ES-NP$_{(BTZ;\ Ce6)}$ are shown in red.

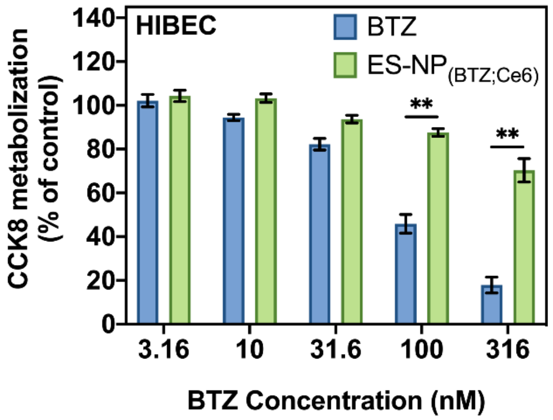

Figure S13: 48 h CCK-8 assays of HIBEC cells treated with different BTZ (equivalent to the dose loaded in ES-NP$_{(BTZ;\ Ce6)}$ or ES-NP$_{(BTZ;\ Ce6)}$ concentrations. The data are represented as mean ± SD (n=3). ***P* < 0.01.

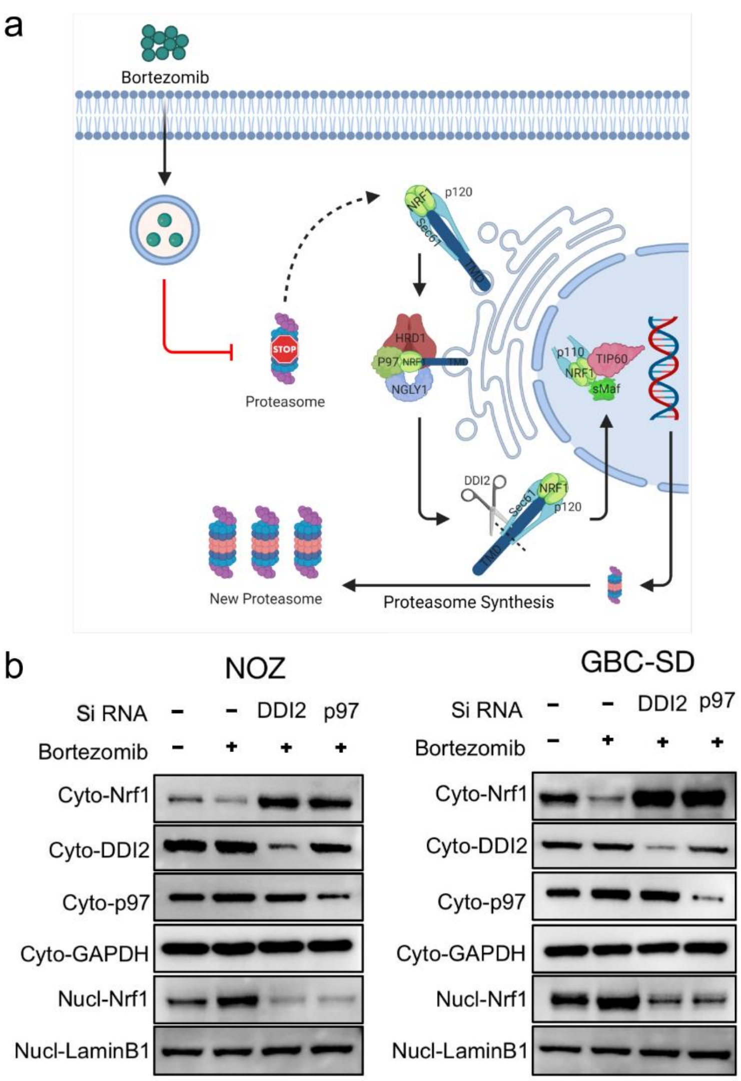

Figure S14: Schematic illustration of molecular mechanism after BTZ administration (a) in GBC cell lines verified by western blotting (b).

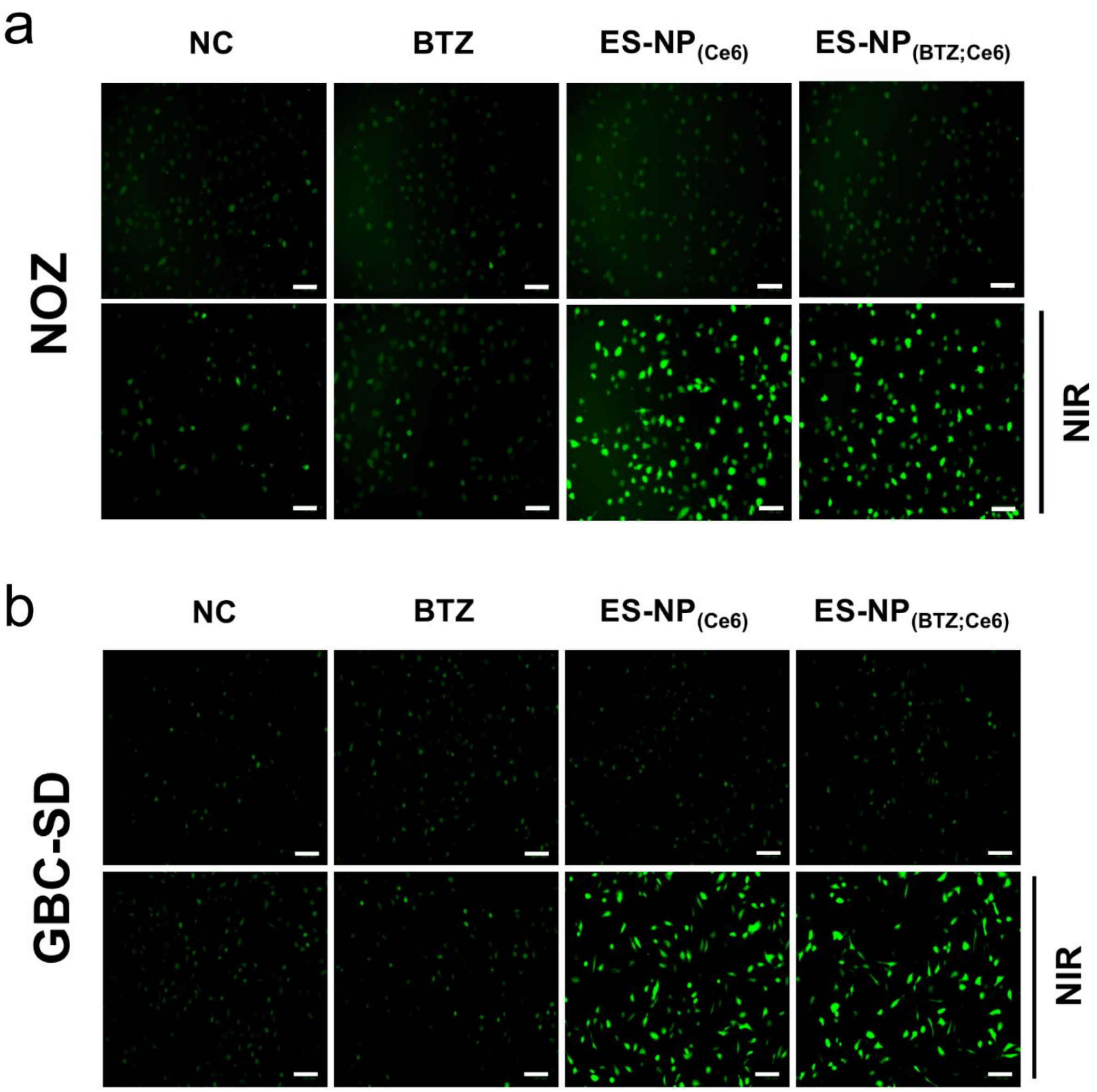

Figure S15: Intracellular ROS observation of a) NOZ or b) GBC-SD cells exposed to normal media, BTZ, ES-NP$_{(Ce6)}$, or ES-NP$_{(BTZ;\ Ce6)}$ with/without 808 nm laser irradiation (2 W cm^{-2}, 5 min with every min interval). Scale bar = 100 µm.

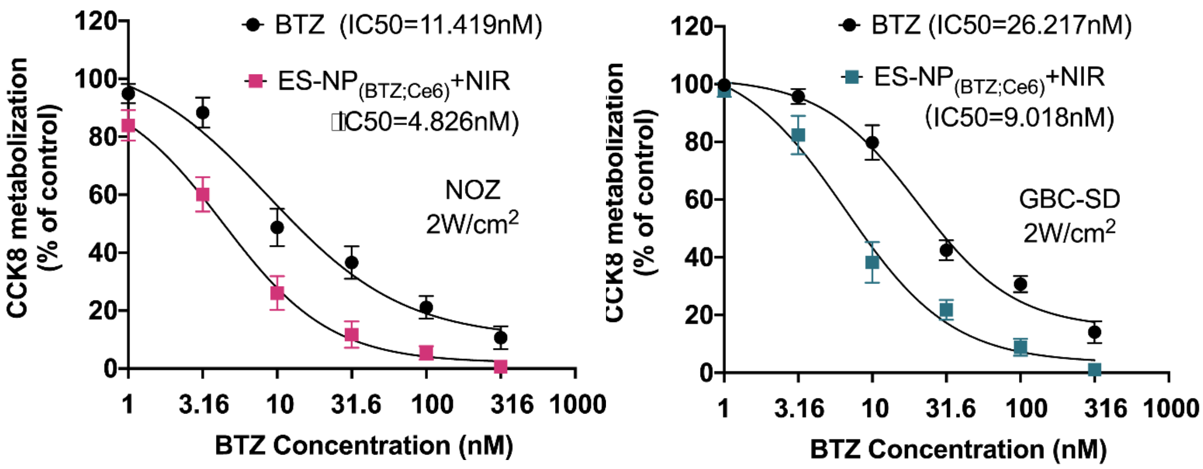

Figure S16: 48 h half-maximal inhibitory concentration (IC50) curve of NOZ and GBC-SD cells exposed to BTZ or ES-NP$_{(BTZ;\ Ce6)}$ with 808 nm laser irradiation (2.0 W cm^{-2}, 5 min with every min interval). The data are represented as mean ± SD (n=3).

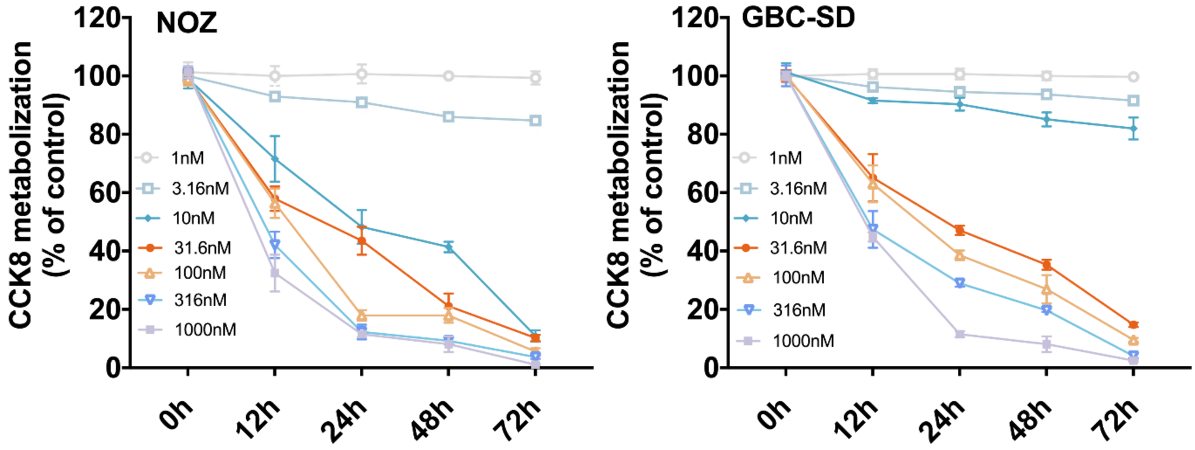

Figure S17: 0-72 h viability of NOZ and GBC-SD cells exposed to different concentrations of BTZ. The data are represented as mean ± SD (n=3).

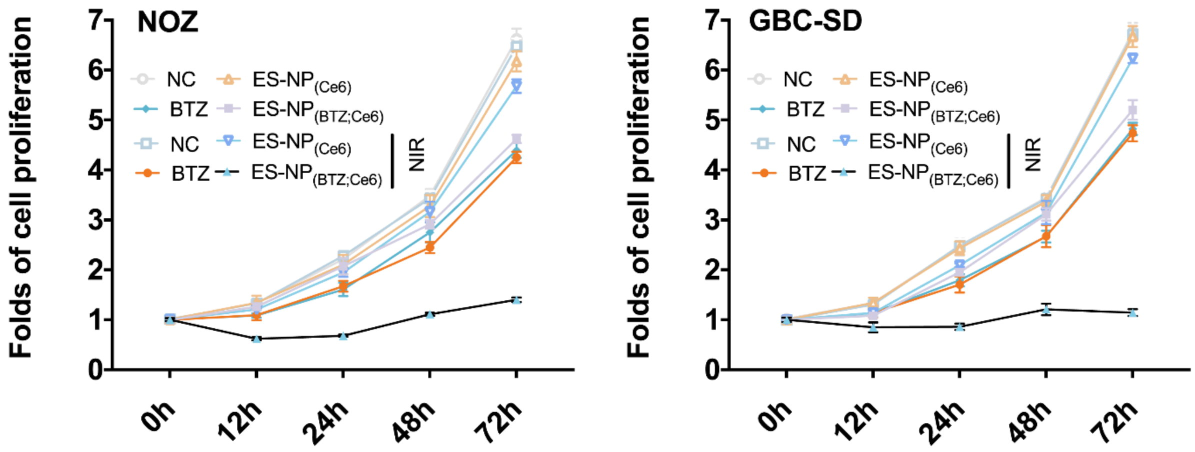

Figure S18: 0-72 h growth curve of NOZ and GBC-SD cells exposed to normal media, BTZ, ES-NP$_{(Ce6)}$, and ES-NP$_{(BTZ;\ Ce6)}$ with and without 808 nm laser irradiation (2.0 W cm^{-2}, 5 min with every min interval). The data are represented as mean ± SD (n=3).

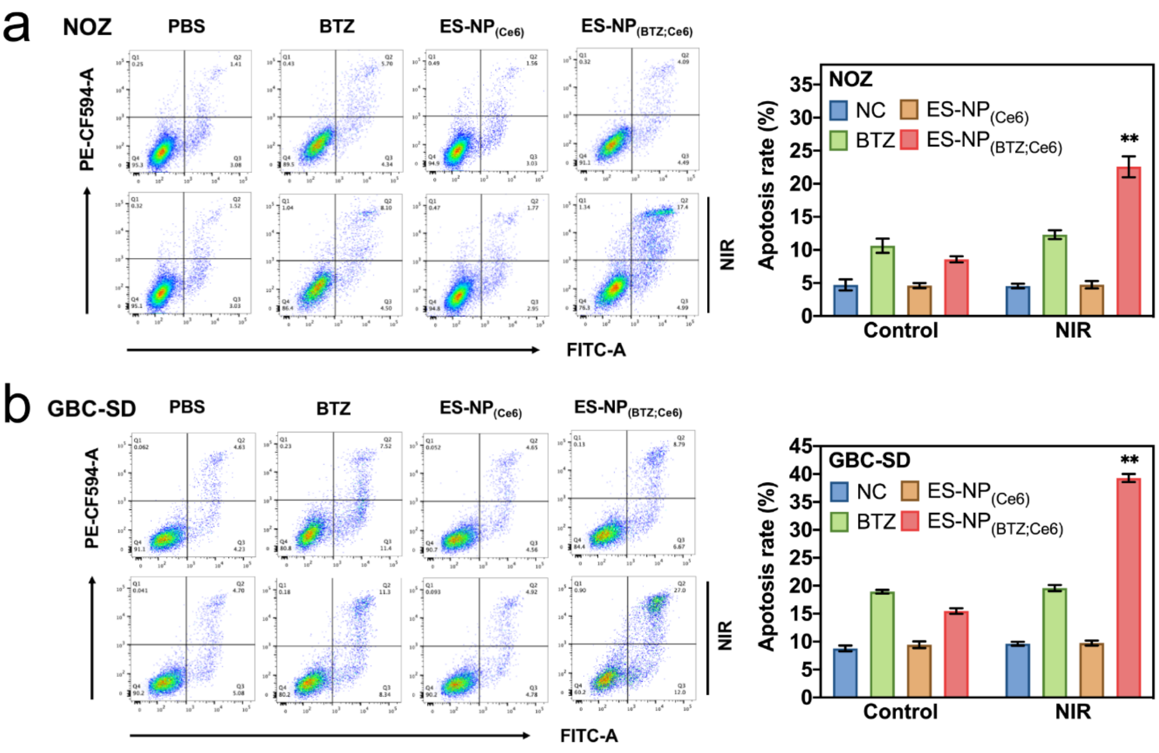

Figure S19: 48 h apoptosis measurement by flow cytometry of a) NOZ or b) GBC-SD cells exposed to normal media, BTZ, ES-NP$_{(Ce6)}$, or ES-NP$_{(BTZ;\ Ce6)}$ with/without 808nm laser irradiation (2 W cm^{-2}, 5 min with every min interval). The data are represented as mean ± SD (n=3). $^{**}P < 0.01$.

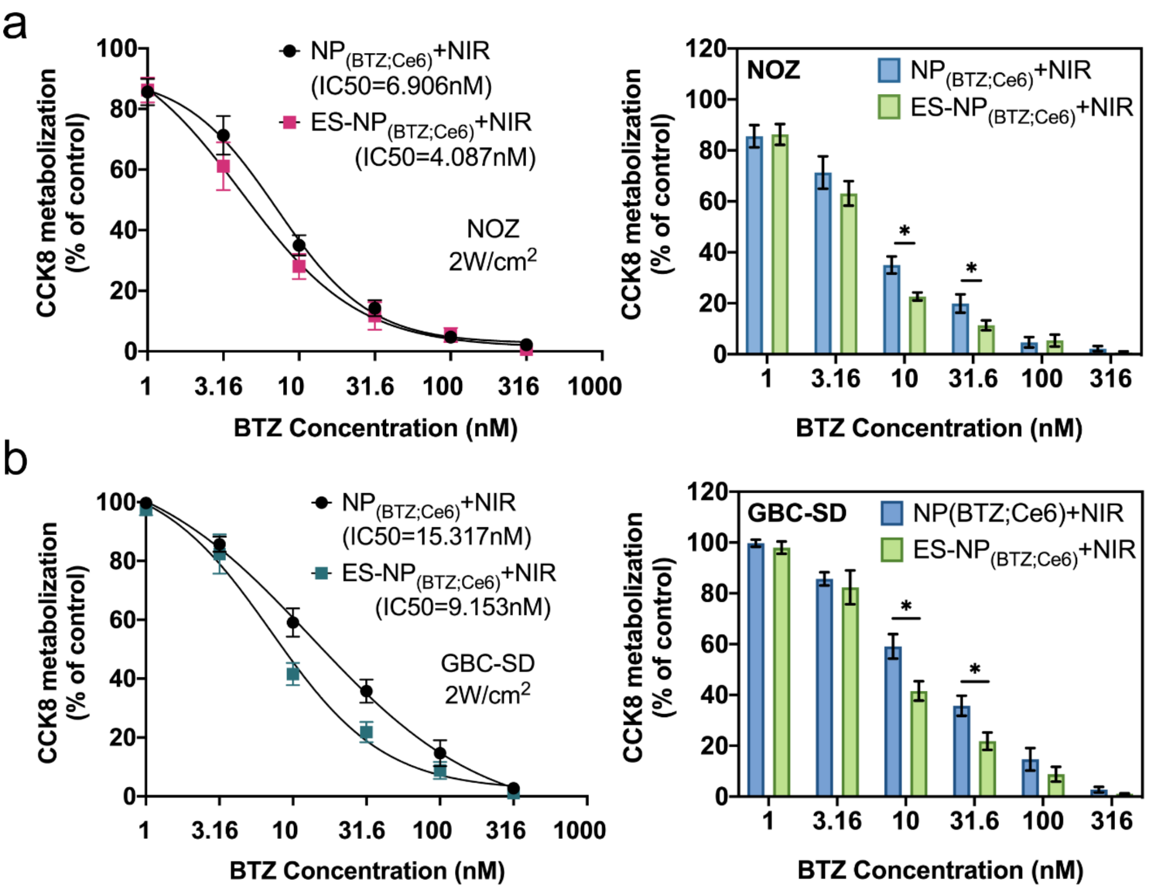

Figure S20: 48 h half-maximal inhibitory concentration (IC50) curve and CCK-8 assays of a) NOZ and b) GBC-SD cells exposed to $NP_{(BTZ;\ Ce6)}$ or $ES\text{-}NP_{(BTZ;\ Ce6)}$ with 808 nm laser irradiation (2.0 W cm^{-2}, 5 min with every min interval). The data are represented as mean ± SD (n=3). *$P < 0.05$.

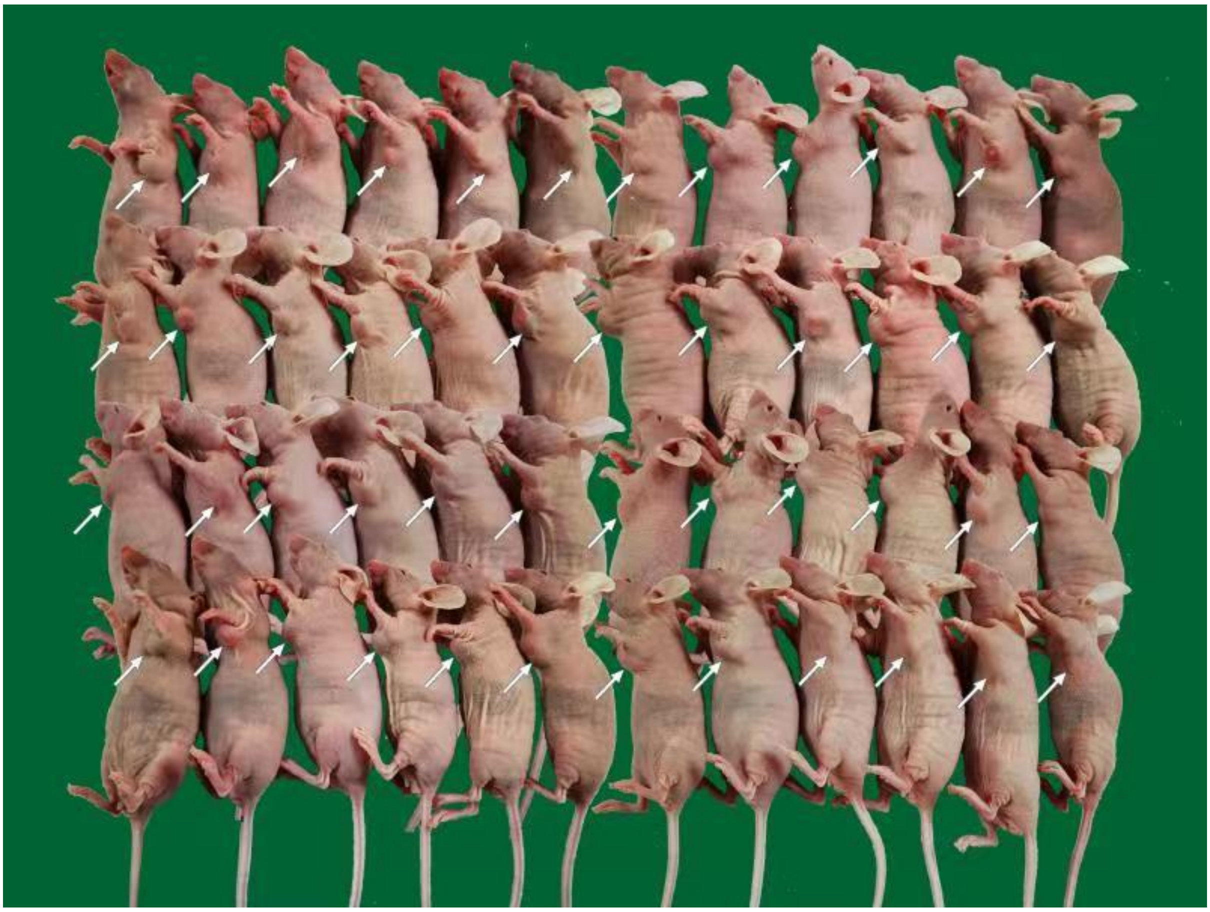

Figure S21: Subcutaneous implant animal model established using NOZ cells.

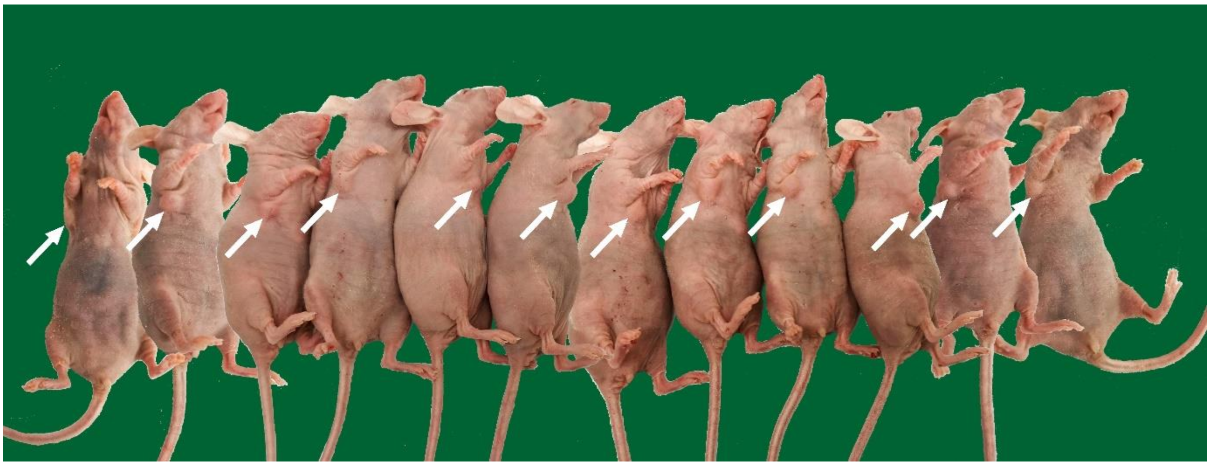

Figure S22: Gallbladder cancer patient-derived xenograft (PDX) animal model established.

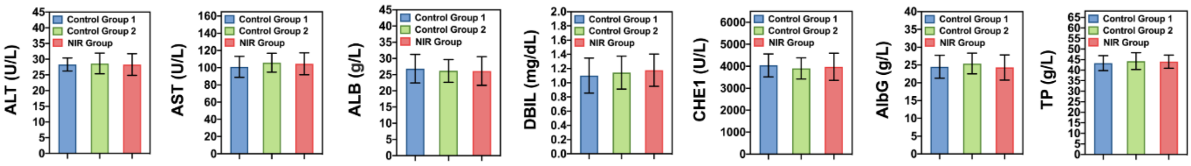

Figure S23: Further hepatotoxicity analysis. Healthy mice were intravenously injected every 3 days for a total 2 times with ES-NP$_{(BTZ;\ Ce6)}$ (Control Group 1), ES-NP$_{(BTZ;\ Ce6)}$ undergoing surgery without intra-abdominal NIR irradiation (Control Group 2), or ES-NP$_{(BTZ;\ Ce6)}$ undergoing surgery with intra-abdominal NIR irradiation (NIR group), and sacrificed at day 20 for blood biochemical analysis. The data are represented as mean ± SD (n = 3 mice per group).
